# Gene Networks of Hyperglycemia, Diabetic Complications, and Human Proteins Targeted by SARS-CoV-2: What Is the Molecular Basis for Comorbidity?

**DOI:** 10.3390/ijms23137247

**Published:** 2022-06-29

**Authors:** Olga V. Saik, Vadim V. Klimontov

**Affiliations:** 1 Laboratory of Endocrinology, Research Institute of Clinical and Experimental Lymphology—Branch of the Institute of Cytology and Genetics, Siberian Branch of Russian Academy of Sciences (RICEL—Branch of ICG SB RAS), Novosibirsk 630060, Russia; klimontov@mail.ru; 2 Laboratory of Computer Proteomics, Federal Research Center Institute of Cytology and Genetics, Siberian Branch of the Russian Academy of Sciences (ICG SB RAS), Novosibirsk 630090, Russia

**Keywords:** diabetes, hyperglycemia, insulin resistance, beta cells, SARS-CoV-2, COVID-19, gene networks, ANDSystem

## Abstract

People with diabetes are more likely to have severe COVID-19 compared to the general population. Moreover, diabetes and COVID-19 demonstrate a certain parallelism in the mechanisms and organ damage. In this work, we applied bioinformatics analysis of associative molecular networks to identify key molecules and pathophysiological processes that determine SARS-CoV-2-induced disorders in patients with diabetes. Using text-mining-based approaches and ANDSystem as a bioinformatics tool, we reconstructed and matched networks related to hyperglycemia, diabetic complications, insulin resistance, and beta cell dysfunction with networks of SARS-CoV-2-targeted proteins. The latter included SARS-CoV-2 entry receptors (ACE2 and DPP4), SARS-CoV-2 entry associated proteases (TMPRSS2, CTSB, and CTSL), and 332 human intracellular proteins interacting with SARS-CoV-2. A number of genes/proteins targeted by SARS-CoV-2 (*ACE2*, *BRD2*, *COMT*, *CTSB*, *CTSL*, *DNMT1*, *DPP4*, *ERP44*, *F2RL1*, *GDF15*, *GPX1*, *HDAC2*, *HMOX1*, *HYOU1*, *IDE*, *LOX*, *NUTF2*, *PCNT*, *PLAT*, *RAB10*, *RHOA*, *SCARB1*, and *SELENOS*) were found in the networks of vascular diabetic complications and insulin resistance. According to the Gene Ontology enrichment analysis, the defined molecules are involved in the response to hypoxia, reactive oxygen species metabolism, immune and inflammatory response, regulation of angiogenesis, platelet degranulation, and other processes. The results expand the understanding of the molecular basis of diabetes and COVID-19 comorbidity.

## 1. Introduction

The coronavirus disease 2019 (COVID-19) pandemic has had a huge impact on morbidity and mortality worldwide. Globally, as of 30 May 2022, there have been 525,467,084 cumulative cases of COVID-19, including 6,285,171 deaths, reported to the WHO [1]. During the outbreak of the epidemic, individuals with diabetes turned out to be one of the most vulnerable cohorts. Though there is no strong evidence that diabetes predisposes to infection with SARS-CoV-2, patients with diabetes demonstrated more severe COVID-19 and higher intensive care unit admission and mortality rates [2,3]. Hyperglycemia has been repeatedly recognized as a risk factor for poor outcomes from COVID-19 in patients with pre-existing diabetes [4,5]. On the other hand, COVID-19 may cause hyperglycemia through the induction of insulin resistance and/or beta cell injury [6,7].

Being completely different in etiology, diabetes and COVID-19 demonstrate a certain parallelism in their mechanisms and organ damage. Specifically, the SARS-CoV-2-induced acute inflammatory response and acute tissue damage that may involve the cardiovascular system, kidneys, and brain correspond to chronic low-grade inflammation, macrovascular disease, chronic kidney disease, neuropathy, and brain changes in diabetes. Hypercoagulability, endothelial dysfunction, oxidative stress, and fibrosis are the common hallmarks of both diseases [8].

In subjects with diabetes, post-COVID-19 syndrome, or long COVID-19, has become a new challenge. The syndrome includes debilitating symptoms and signs that develop during or after an infection consistent with COVID-19, persist for more than 12 weeks, and cannot be explained by an alternative diagnosis [9]. Clinical manifestations of post-COVID-19 syndrome are very diverse, with weakness, general malaise, fatigue, concentration impairment, and breathlessness being the most common symptoms reported [10]. Although the hypothesis that patients with diabetes are more likely to develop post-COVID-19 syndrome had not been confirmed [11], the association between the two disorders indicates that they may be mutually aggravating [12]. Taken into account SARS-CoV-2-induced pathophysiological changes, such as dysregulation of inflammatory and immune response, oxidative stress, hypercoagulability, capillary damage, and tissue hypoxia [13], as well as instability in glycemic control [14], one can assume that long COVID-19 may contribute to the progression of diabetic complications. Identification of the molecular mechanisms of metabolic disorders and organ damage in subjects with diabetes during and after COVID-19 is an urgent task.

According to the Human Protein Atlas [15], human proteins related to SARS-CoV-2 can be divided into three groups: SARS-CoV-2 entry receptors, SARS-CoV-2 entry associated proteases, and intracellular SARS-CoV-2 interacting proteins (https://www.proteinatlas.org/humanproteome/sars-cov-2, accessed on 10 February 2022). The most known SARS-CoV-2 entry receptor is angiotensin-converting enzyme 2 (ACE2) [16]. Dipeptidyl peptidase-4 (DPP4) is also involved in the entry [17]. SARS-CoV-2 entry-associated proteases include transmembrane protease, serine 2 (TMPRSS2) [18], cathepsin B (CTSB) [19], and cathepsin L (CTSL) [20]. A set of 332 human intracellular proteins interacting with SARS-CoV-2 was identified by Gordon et al. [21].

In modern biology and medicine, gene network analysis is considered a useful tool for studying the molecular mechanisms of physiological processes and diseases [22]. Thus, in this study, we matched the molecular networks of hyperglycemia and diabetic complications with the networks of human proteins related to SARS-CoV-2. To build molecular (gene) networks, we used the ANDSystem, a bioinformatics tool that performs text-mining of PubMed/Medline-indexed publications. The ANDSystem reconstructs molecular networks as data graphs with nodes including molecules and edges showing the types of connections between nodes [23,24]. The system can be used for the analysis of molecular mechanisms of human diseases and their associations [25,26]. Recently, we applied this tool to reconstruct and analyze the gene networks of diabetic complications, glucose variability, and hypoglycemia [27,28].

The aim of this study was to find key molecules and biological processes that mediate the development of SARS-CoV-2-induced metabolic disorders and tissue damage in patients with diabetes by matching the gene networks of hyperglycemia, diabetic complications, insulin resistance, and beta cell dysfunction with the networks of human proteins targeted by SARS-CoV-2.

## 2. Results and Discussion

### 2.1. Network Associated with Hyperglycemia

**Figure 1:**
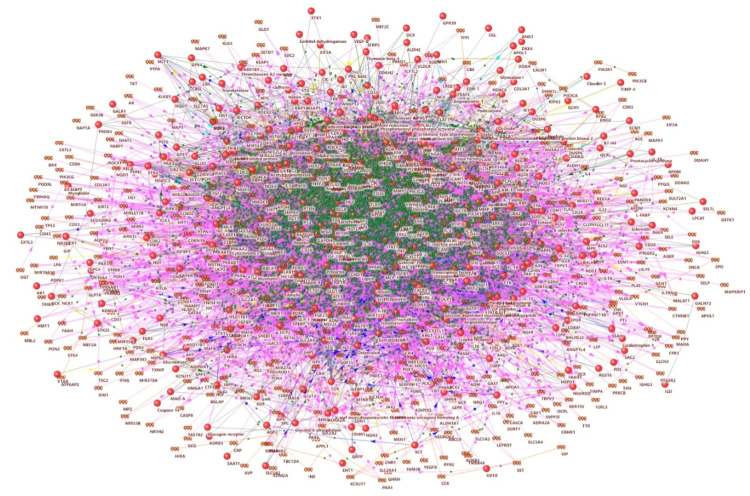
Molecular network associated with hyperglycemia.

In the first step, we updated a previously reconstructed gene network associated with hyperglycemia [27]. The updated network included 430 genes/proteins and 46,855 interactions between them (Figure 1, Table S1).

**Figure 2:**
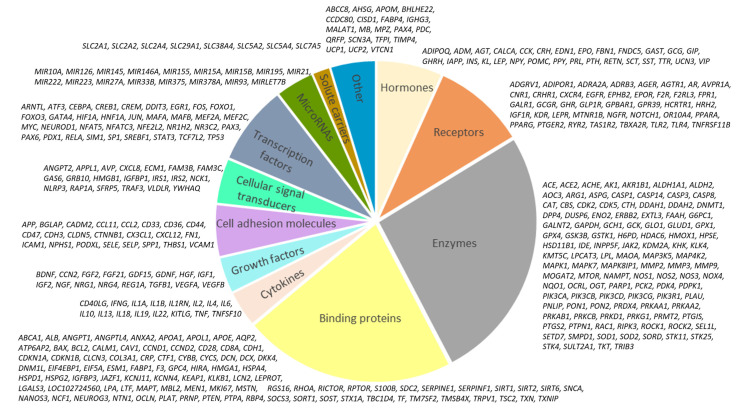
Molecules in the hyperglycemia-associated network.

The functional diversity of the molecules included in the network is presented in Figure 2. Among the nodes of the network, hormones, receptors, enzymes, binding proteins, cytokines, growth factors, cell adhesion molecules, receptors, transcription factors, signal transducers, solute carriers, microRNAs, and other molecules were identified.

**Figure 3:**
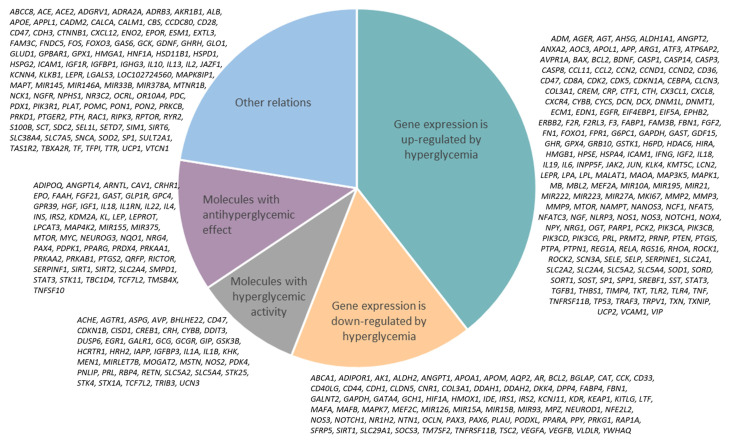
Types of associations between genes and HG in the hyperglycemia network.

The variants of the associations between high glucose (HG) and identified genes are presented in Figure 3. It was shown that hyperglycemia upregulates the expression of 179 genes and downregulates 75 genes. On the other hand, 44 molecules contribute to hyperglycemia development and 54 demonstrate antihyperglycemic activity.

Insulin (*INS*), interleukin-6 (*IL6*), tumor protein P53 (*TP53*), mitogen-activated protein kinase 1 (*MAPK1*), tumor necrosis factor (*TNF*), glyceraldehydes-3-phosphate dehydrogenase (*GAPDH*), epidermal growth factor receptor (*EGFR*), signal transducer and activator of transcription 3 (*STAT3*), matrix metalloproteinase-9 (*MMP9*), and leptin (*LEP*) genes were the central hubs of the hyperglycemia-associated network with the highest betweenness centrality values (Table S2). The betweenness centrality reflects the involvement of a node (molecule) in signal transduction through a network. This measure is calculated based on the number of the shortest paths connecting all pairs of nodes in the network that go through the analyzed node. The high value of betweenness centrality means that the node is a key player or a “bridge” between different parts of a network [29]. Products of the identified genes with the highest betweenness centrality values participate in the regulation of glucose and lipid metabolism (*INS*, *GAPDH*, *LEP*), cell cycle and apoptosis (*TP53*, *MAPK1*, *STAT3*), immune and inflammatory response (*IL6*, *TNF*, *MMP9*), cell proliferation, differentiation, and survival (*INS*, *LEP*, *EGFR*, *STAT3*). The role of these molecules in glucose metabolism and hyperglycemia-related biochemical abnormalities have been discussed previously [27,30].

We have also identified the genes with the highest crosstalk specificity (CTS) values. The CTS is calculated as a number of neighbors of a particular node (molecule) in a network divided by the number of all neighbors of the node in the global human gene network of the ANDSystem. A higher CTS value means that a node is closely and specifically related to a studied network [24,26,27,28]. The highest crosstalk specificity (CTS) values (Table S3) were demonstrated by uncoupling protein 2 (*UCP2*), hydroxisteroid 11-beta dehydrohenase-1 (*HSD11B1*), interleukin-19 (*IL19*), fatty acid binding protein 1 (*FABP1*), pyroglutamylated RFamide peptide (*QRFP*), resistin (*RETN*), solute carrier family 2 member 2 (*SLC2A2*), leptin receptor overlapping transport (*LEPROT*), endothelial cell-specific molecule 1 (*ESM1*), and cholecystokinin (*CCK*) genes.

Mitochondrial uncoupling protein 2 (*UCP2*) is a member of the mitochondrial anion carrier proteins family. It is involved in the separation of oxidative phosphorylation from ATP synthesis with heat release. Hyperglycemia was shown to increase UCP2 levels [31,32] that could be a compensatory effect to the elevated reactive oxygen species generation [33]. UCP2 was shown to negatively regulate insulin secretion. It was proposed as a key factor in beta cell glucose sensing, and a critical interlayer connecting obesity, beta cell dysfunction, and type 2 diabetes [34]. Hydroxysteroid 11-beta dehydrogenase 1 (*HSD11B1*) catalyzes the bidirectional reaction of the conversion of cortisol to cortisone. The activity of this enzyme is impaired in hyperglycemic conditions [35]. Interleukin-19 (*IL19*) is a cytokine that can bind the IL20 receptor complex and lead to STAT3 activation. It is also involved in apoptosis induction and inflammatory response. Long-term hyperglycemia may increase *IL19* expression in endothelial cells, resulting in local inflammation and accelerated endothelial damage [36]. Fatty acid-binding protein 1 (*FABP1*) binds long-chain fatty acids, bile acids, and other hydrophobic ligands; it is involved in fatty acid uptake, transport, and metabolism. The expression of *FABP1* increases during hyperglycemia [37]. Pyroglutamylated RFamide peptide (*QRFP*) is a precursor of the members of the RFamide neuropeptides family, some of which are able to regulate blood pressure, reproduction, and food intake. The 26RFa product of *QRFP* reduces glucose-induced hyperglycemia and increases insulin sensitivity and insulin levels [38]. Resistin (*RETN*) is an adipokine that reduces insulin sensitivity, enhances hepatic gluconeogenesis, and increases lipolysis and serum-free fatty acid levels [39]. Solute carrier family 2 member 2 (*SLC2A2*) is an integral plasma membrane glycoprotein of the liver, islet beta cells, intestine, and kidney epithelium that mediates facilitated bidirectional glucose transport. It is also discussed as a glucose sensor with low glucose affinity. The SLC2A2 level is increased in hyperglycemia and decreased in hyperinsulinemia [40]. Leptin receptor overlapping transcript (*LEPROT*) is involved in the expression of growth hormone and leptin receptors, which are associated with hyperglycemia [41]. Endothelial cell-specific molecule 1 (*ESM1*) is regulated by cytokines and could have a role in the endothelial dysfunction [42]. Cholecystokinin (*CCK*) is further processed to multiple protein products such as cholecystokinin-8, -12, -33 peptide hormones, which are able to regulate gastric acid secretion and food intake. Cholecystokinin regulates postprandial hyperglycemia [43]; hyperglycemia, in its turn, could influence the satiating effect of cholecystokinin [44].

The Gene Ontology (GO) enrichment analysis performed by DAVID [45] revealed glucose homeostasis, inflammatory response, response to hypoxia, regulation of cell proliferation, angiogenesis and apoptosis, aging, and response to drugs among the most overrepresented biological processes associated with hyperglycemia (Table 1, Table S4).

These data demonstrate that hyperglycemia is associated with the upregulation or downregulation of a large number of genes whose products are involved in a wide range of physiological and pathophysiological processes. This may lead to an abnormal response to other stressor factors, including a viral infection.

### 2.2. Networks Associated with Diabetic Complications, Insulin Resistance, and Beta-Cell Dysfunction

In this work, we operated on gene networks associated with cardiovascular disease (CVD), diabetic nephropathy, diabetic retinopathy, and diabetic neuropathy that have been reconstructed and described previously [27,28]. These networks include 494, 685, 424, and 130 genes/proteins, respectively (Table S5).

Gene networks associated with insulin resistance and beta-cell dysfunction were built using the ANDSystem. These networks contain 1452 and 72 genes/proteins, respectively (Tables S6 and S7).

### 2.3. Networks of Human Proteins Related to SARS-CoV-2

#### 2.3.1. SARS-CoV-2 Entry Receptors

##### ACE2-Related Network

ACE2, a carboxypeptidase, mediates vasodilation by cleaving angiotensin II and takes part in the negative regulation of the renin–angiotensin system. It was postulated that ACE2 is the main target of SARS-CoV-2 that supports its entrance into the human cells [16]. Interestingly, the loss of ACE2 in mice results in alterations in glucose tolerance and reduces the first phase of insulin secretion [46]. In *db*/*db* mice, the progression of type 2 diabetes was accompanied by ACE2 depletion; ACE2 restoration improved glycemia [47].

According to the ANDSystem, ACE2 directly interacts with 147 genes/proteins in the global human network (Table S8). Among them, 34 are participants of the hyperglycemia network (Table 2). The enrichment of the hyperglycemia network with genes/proteins interacting with ACE2 was statistically significant (*p*-value < 10^−24^).

It could be assumed that the interaction between SARS-CoV-2 and ACE2 disrupts the function of ACE2 and activity of ACE2-interacting molecules (Table 2). Among these molecules, of greatest interest are those that are upregulated by HG and downregulated by ACE2. This group includes angiopoietin-2 (*ANGPT2*), monocyte chemoattractant protein 1 (*CCL2*), connective tissue growth factor (*CCN2*), high-mobility group protein B1 (*HMGB1*), inter-cellular adhesion molecule 1 (*ICAM1*), vascular cell adhesion molecules 1 (*VCAM1*), miRNA-21 (*MIR21*), matrix metalloproteinase-9 (*MMP9*), and signal transducer and activator of transcription 3 (*STAT3*). As the binding of viral particles to ACE2 could lead to attenuation of ACE2’s ability to downregulate these genes/proteins, the upregulation can be assumed. In diabetes, HG can also upregulate the expression of these genes. This double effect can significantly activate the synthesis of the products of these genes, creating a background for comorbidity.

**Figure 4:**
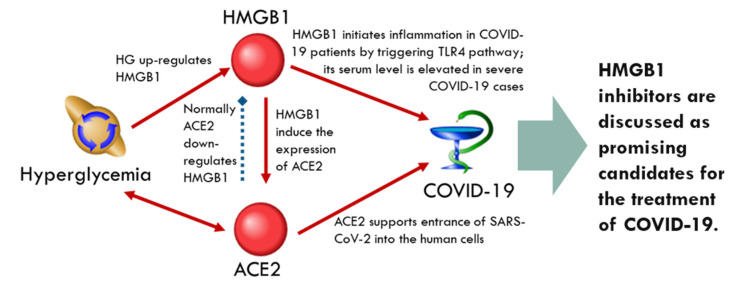
Positive feedback loop that involves HMGB1 in ACE2-related network. The red arrows correspond to the up-regulation and the blue arrow corresponds to the down-regulation.

Some clinical evidence supports this assumption. It was shown that angiopoietin-2 levels were increased in COVID-19 patients and demonstrated relations with the disease severity, hypercoagulation, and mortality [48,49]. Monocyte chemoattractant protein 1 was also linked to COVID-19 severity; it was upregulated during the early phase of SARS-CoV-2 infection and increased further at the late stages in fatal cases [50]. Connective tissue growth factor is considered a fibrotic biomarker [51]. The serum levels of ICAM-1 and VCAM-1 were elevated in patients with COVID-19, especially in severe cases; the molecules demonstrated relations with coagulation disorders [52]. Enhanced ICAM-1 concentration was an independent predictor of mortality in COVID-19 [53]. The fibrosis-associated miRNA-21 was increased in the acute phase of COVID-19 infection and its upregulation turned out to be a predictor of chronic myocardial damage and inflammation in COVID-19 survivors [54]. The levels of MMP-9 were higher in COVID-19 patients and were considered an early indicator of respiratory failure and mortality [55,56]. It was supposed that the hyperactivation of STAT3 participates in the induction of a cytokine storm, the suppression of the antivirus interferon response, M2 macrophage polarization, and lung fibrosis and thrombosis in COVID-19 [57]. The HMGB-1 is thought to initiate inflammation in COVID-19 patients by triggering TLR4 pathway [58]; its serum level is elevated in severe COVID-19 cases [59]. It is also important to mention that HMGB1 itself is able to induce the expression of ACE2 in alveolar epithelial cells [58,59], forming a positive feedback loop in a gene network and amplifying the pathological signals during COVID-19 and hyperglycemia (Figure 4). Accordingly, HMGB1 inhibitors were discussed as promising candidates for the treatment of COVID-19 [59].

Among other components of the network, sirtuin 1 (*SIRT1*), angiotensin II receptor type 1 (*AGTR1*), apolipoprotein E (*APOE*), and ACE (*ACE*) are also worth mentioning. Hyperglycemia is known to induce the downregulation of *SIRT1* [60]; in turn, sirtuin 1 downregulates ACE2 expression [61]. Thus, it could be suggested that hyperglycemia can induce ACE2 by blocking its repressor, leading to the more effective entrance of viral particles into the cells. Indeed, a deficiency of *SIRT1* was linked with the hyperinflammatory response and increased mortality in COVID-19 [62,63]. It was shown that *AGTR1* is normally downregulated by ACE2 [64] and possesses hyperglycemic activity [65]. The A/A genotype of *rs5183* SNP in the *AGTR1* gene was associated with higher hospitalization risk in patients with COVID-19 and comorbidities [66]. The apolipoprotein E ε4 allele (APOE4) was associated with ACE2 reduction [67] and blood glucose level [68]. It was linked to increased susceptibility to SARS-CoV-2 infection, severe COVID-19 course, post-COVID mental fatigue, and COVID-19 mortality [69,70]. ACE is able to downregulate ACE2 [71] and, in turn, it is downregulated by ACE2 [72]. This reciprocal regulation constitutes a loop in the gene network that modulates the balance between vasoconstriction and vasodilation. *ACE* rs4646994 SNP was shown to increase the risk of COVID-19 infection [73].

ACE2 is known as a critical participant in cardiovascular homeostasis and its altered expression is associated with CVD [74]. The inhibition of ACE2 accelerates diabetic kidney injury and renal ACE2 is downregulated in diabetic nephropathy [75]. The loss of ACE2 aggravates diabetic retinopathy by promoting bone marrow dysfunction [76]. The absence of ACE2 resulted in exaggerated glucose intolerance with insulin resistance [77].

The genes/proteins interacting with ACE2 were found in the gene networks of diabetes complications: there were 44 genes/proteins in the CVD network, 9 in the diabetic neuropathy network, 51 in the diabetic nephropathy network, 40 in the diabetic retinopathy network, 75 in the insulin resistance network, and 4 in the beta-cell dysfunction network. All of these networks were enriched by ACE2-interacting genes/proteins with statistically significant *p*-values less than 10^−34^, 10^−6^, 10^−36^, 10^−32^, 10^−45^, and 0.002, respectively.

According to the GO enrichment analysis, these genes are involved in the regulation of cell migration, gene expression, cell proliferation, phosphatidylinositol 3-kinase signaling, apoptosis, response to hypoxia and lipopolysaccharide, nitric oxide signaling, and the regulation of vascular endothelial cell proliferation (Table 3). Moreover, there were inflammatory response, blood vessel remodeling, angiogenesis, regulation of vascular tone and blood pressure, fatty acid and glucose homeostasis, and aging (Table S9).

##### DPP4-Related Network

DPP4 is an enzyme involved in glucose and insulin metabolism, as well as in immune regulation. It is thought to be a functional receptor of human coronavirus; it can directly bind with the S protein of SARS-CoV-2 [17]. In the global human gene network of the ANDSystem, DPP4 is linked with 251 genes/proteins (Table S10), and 48 of them are also involved in the hyperglycemia network. The enrichment of the hyperglycemia network with genes/proteins linked with DPP4 was statistically significant (*p*-value < 10^−30^).

The types of associations of the genes/proteins from hyperglycemia-related and DPP4-related networks with HG and DPP4 are presented in Table 4. Some identified genes, including *CCL11*, *FGF2*, *HMGB1*, and *MMP9*, are upregulated by HG [78,79,80,81] and downregulated by DPP4 [82,83,84,85]. In COVID-19, the ability of DPP4 to downregulate these genes could be attenuated. In COVID-19 patients, including those with long COVID with cognitive symptoms, the level of eotaxin-1 encoded by *CCL11* was significantly increased [86,87]. Intradermal administration of eotaxin-1 upregulated *DPP4* in rats [88], constituting a regulatory loop. The elevation of the serum level of fibroblast growth factor 2 (*FGF2*) in COVID-19 patients was closely associated with disease severity and admission to an intensive care unit [89]. The clinical significance of MMP-9 and HMGB1 in COVID-19 was mentioned above [55,56,59].

**Figure 5:**
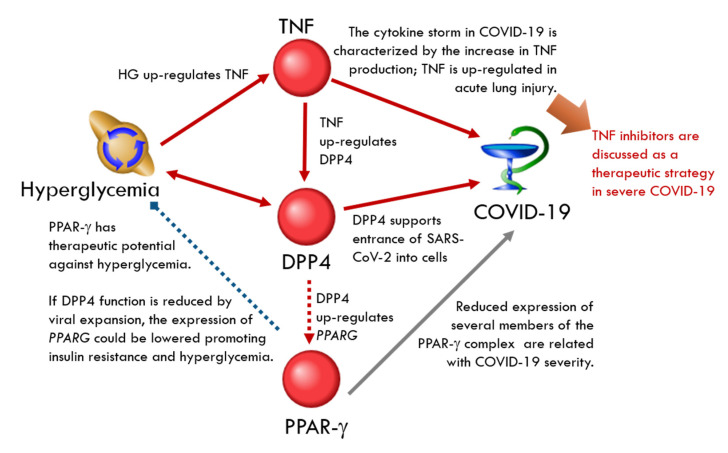
The feedback loop in DPP4-related network involves TNF and PPAR-γ. The red arrows correspond to the up-regulation, the blue arrow corresponds to the down-regulation and the grey arrow corresponds to the association.

Tumor necrosis factor (TNF) and peroxisome proliferator-activated receptor gamma (PPARγ) could be important players in the hyperglycemia–COVID-19 relationship (Figure 5). Hyperglycemia induces the overproduction of TNF [90] and circulating plasma DPP4 levels are significantly upregulated by this factor [91]. The cytokine storm in COVID-19, associated with the severity of the disease, is characterized by the increase in TNF production; TNF is upregulated in acute lung injury and facilitates SARS-CoV-2 interaction with ACE2. Accordingly, TNF inhibitors were discussed as a therapeutic strategy in severe COVID-19 [92].

PPAR-γ has therapeutic potential against hyperglycemia [93], and its expression is increased by DPP4 [94]. In lung biopsies from patients with COVID-19, the gene enrichment patterns were similar to that of *PPARG*-knockout macrophages. There was a relation between the disease severity and reduced expression of several members of the PPARγ complex [95]. If DPP4 function is reduced by viral expansion, the expression of *PPARG* could be lowered, promoting insulin resistance and hyperglycemia.

DPP4 and the genes/proteins interacting with it were also found in the analyzed gene networks of diabetes complications. Sixty genes/proteins were identified in the CVD network, 23 in the network of diabetic neuropathy, 79 in the diabetic nephropathy network, 56 in the diabetic retinopathy network, 126 in the insulin resistance network, and 6 in the beta-cell dysfunction network. All of these networks were enriched by DPP4-interacting genes/proteins with statistically significant *p*-values less than 10^−42^, 10^−19^, 10^−54^, 10^−40^, 10^−76^, and 0.0003, respectively.

The GO enrichment analysis revealed the response to hypoxia, regulation of ERK1 and ERK2 cascade, phosphatidylinositol 3-kinase signaling, interleukin-8 production, lipid storage and smooth muscle cell proliferation, aging, cellular response to lipopolysaccharide, and acute-phase response among principal biological processes regulated by the genes linked to DPP4, hyperglycemia, and diabetic complications (Table 5). The regulation of insulin secretion, glucose homeostasis, regulation of MAPK cascade, vasodilation, inflammatory response, and regulation of cytokine production added to the list of overrepresented processes (Table S11).

#### 2.3.2. SARS-CoV-2 Entry-Associated Protease Receptors

##### TMPRSS2-Related Network

**Figure 6:**
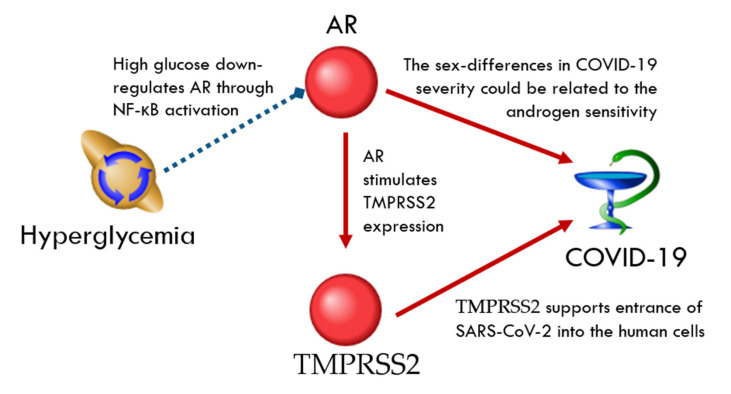
The regulation loop in the TMPRSS2-related network involves AR. The red arrows correspond to the up-regulation and the blue arrow corresponds to the down-regulation.

TMPRSS2, a serine protease, is involved in SARS-CoV-2 host cells entry by S protein priming [18]. In the ANDSystem global human gene network, TMPRSS2 was linked to 52 genes/proteins (Table S12). Among these molecules, the androgen receptor (AR) was the only one that was also present in the hyperglycemia network. It was shown that high glucose downregulates AR mRNA and protein levels in LNCaP cells through NF-κB activation [96]. In turn, AR stimulates TMPRSS2 expression [97], facilitating the SARS-CoV-2 entry (Figure 6). It was postulated that the sex differences in COVID-19 severity could be related to androgen sensitivity [98].

Some genes/proteins linked with TMPRSS2 were also present in the networks of diabetic complications and diabetes-related impaired insulin sensitivity and insulin secretion. Only 3 genes/proteins were found in the CVD network, 4 in the network of diabetic neuropathy, 10 in the diabetic nephropathy network, 6 in the diabetic retinopathy network, 12 in the insulin resistance network, and 1 in the network of beta-cell dysfunction. Except for the gene networks of CVD and beta-cell dysfunction, there was some enrichment of the analyzed networks with the TMPRSS2-interacting genes/proteins with *p*-values less than 0.0004 for diabetic neuropathy, 10^−5^ for diabetic nephropathy, 0.0008 for diabetic retinopathy, and 0.0003 for the insulin resistance network.

According to the obtained results, the role of TMPRSS2 in the crosstalk between diabetes-related metabolic disorders, diabetic complications, and COVID-19 seems to be modest.

##### CTSB-Related Network

Cathepsin B (*CTSB*), a cysteine protease, facilitates the entry of SARS-CoV-2 into the target host cells by the activation of the viral surface protein S [19]. CTSB was directly linked to 329 genes/proteins in the global human network reconstructed by the ANDSystem (Table S13). Among these molecules, 48 were the components of the hyperglycemia-related network (Table 6). The enrichment of hyperglycemia network with genes/proteins interacting with *CTSB* was statistically significant (*p*-value < 10^−25^).

**Figure 7:**
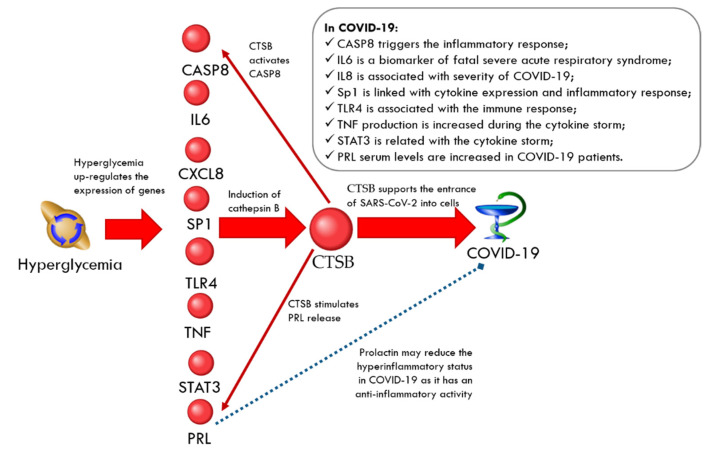
The regulation loop in the CTSB-related network involves CASP8, IL6, CXCL8, SP1, TLR4, TNF, STAT3, and PRL. The red arrows correspond to the up-regulation and the blue arrow corresponds to the down-regulation.

The associations of the gene/proteins from hyperglycemia-related and cathepsin B (CTSB)-related networks with HG and cathepsin B are presented in Table 6. As shown in Figure 7, the expression of caspase 8 (*CASP8*), interleukin-6 (*IL6*), interleukin-8 (*CXCL8*), Sp1 transcription factor (*SP1*), toll-like receptor 4 (*TLR4*), *TNF*, *STAT3*, and prolactin (*PRL*) are upregulated by hyperglycemia [99,100,101,102,103,104,105,106] and are known to induce cathepsin B [107,108,109,110,111,112,113,114]. In COVID-19, the inflammatory response and cell death are triggered via caspase 8 activation [115] and cathepsin B is able to activate this enzyme [116]. Interleukin-8 participates in the signaling axis, determining the severity of COVID-19 [117]. Interleukin-6 was proposed as a biomarker for the development of fatal severe acute respiratory syndrome in COVID-19 [118]. It was reported that prolactin serum levels are increased in COVID-19 patients [119]. Cathepsin B stimulates prolactin release [120]. The excess of prolactin can contribute to hyperglycemia by the reduction of insulin sensitivity [121]. On the other hand, prolactin may reduce the hyperinflammatory status in COVID-19 as it has an anti-inflammatory activity [119]. The Sp1 transcription factor could be linked with cytokine expression and the inflammatory response in COVID-19 via miR-155-5p [122]. STAT3 hyperactivation is related to the cytokine storm in COVID-19 [57]. TLR4 was discussed as a prime regulatory factor associated with the immunity and pathogenesis of SARS-CoV-2 infection [123].

Some other genes/proteins listed in Table 6 have been studied in COVID-19 [124,125,126,127,128,129,130,131,132,133,134,135,136,137,138,139,140,141,142,143,144,145,146,147]. It was shown that serum brain-derived neurotrophic factor (*BDNF*) is associated with poor prognosis of the disease [124]. The activation of caspase 1 (*CASP1*) was related to a severe course of COVID-19 [125]. In red blood cells obtained from COVID-19 patients, the levels of caspase-3/7 were elevated [126] and the *CASP3* gene was a prognostic marker for COVID-19 severity [127]. The level of interleukin-18 (*IL18*) was significantly higher in patients with severe COVID-19 than in those with milder disease [128]. A dramatic and early rise in IL-10 was observed in severe SARS-CoV-2 infection [129]. It was reported that a TGFB1-related chronic immune response is induced in severe COVID-19 [130]. The levels of anti-ANXA2 antibodies predicted mortality among hospitalized COVID-19 patients [131,132].

It was found that cathepsin B participates in the conversion of proinsulin to insulin. It is also involved in some diabetic complications, including CVD [148,149]. The down-regulation of *CTSB* suppresses autophagy and promotes apoptosis contributing to the development of proliferative diabetic retinopathy [150]. The insulin resistance causes the downregulation of *CTSB* [151].

The genes/proteins interacting with *CTSB* were found in the analyzed gene networks: 59 genes/proteins were revealed in the CVD network, 23 in the diabetic neuropathy, 84 in the diabetic nephropathy, 58 in the diabetic retinopathy, 124 in the insulin resistance, and 12 in the beta-cell dysfunction network. All of these networks were enriched by CTSB-interacting genes/proteins with statistically significant *p*-values (less than 10^−32^, 10^−16^, 10^−48^, 10^−35^, 10^−56^, and 10^−8^ respectively).

According to the GO enrichment analysis, the genes linked with *CTSB* and incorporated in the discussed networks were involved in the regulation of cell proliferation, gene expression, protein phosphorylation, and interleukin-8 production, protein kinase B and lipopolysaccharide-mediated signaling pathways, response to drug, and apoptosis (Table 7). The insulin secretion, inflammatory response, regulation of cytokine production, and response to hypoxia were also overrepresented (Table S14).

##### CTSL-Related Network

Cathepsin L, a lysosomal cysteine proteinase encoded by the *CTSL* gene, was shown to cleave the SARS-CoV-2 spike protein and enhance virus entry. Its circulating level is elevated in SARS-CoV-2 infection and it is positively correlated with the disease course and severity [20]. In the global human network estimated by the ANDSystem, *CTSL* is directly linked to 212 genes/proteins (Table S15). Among them, 22 molecules were also revealed in the hyperglycemia network (Table 8). The enrichment of the hyperglycemia network with genes/proteins interacting with *CTSL* was statistically significant (*p*-value < 10^−8^).

**Figure 8:**
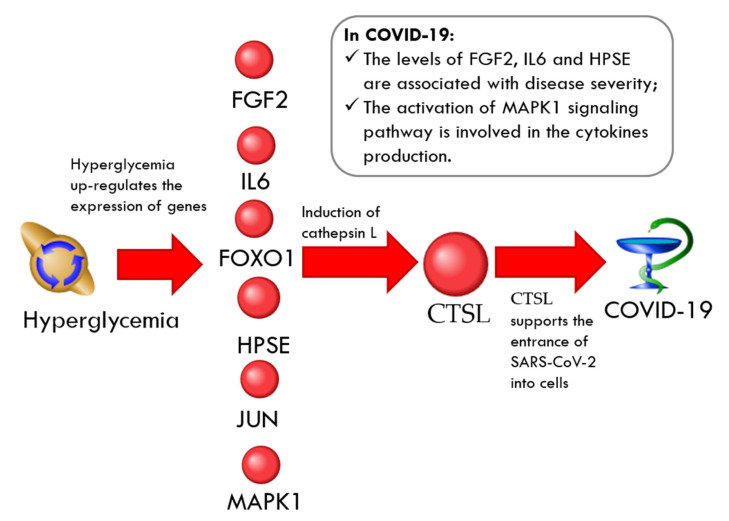
The regulation loop in the CTSL-related network involves FGF2, IL6, FOXO1, HPSE, JUN, and MAPK1.

As shown in Table 8, *FGF2*, *IL6*, *FOXO1*, *HPSE*, *JUN*, and *MAPK1* are upregulated by hyperglycemia [79,101,152,153,154,155] and can activate the cathepsin L [156,157,158,159,160,161]. For some of these molecules, there is clinical evidence of an association with COVID-19 (Figure 8). Specifically, the levels of fibroblast growth factor 2 (*FGF2*), interleukin-6 (*IL6*), and heparanase (*HPSE*) were associated with COVID-19 disease severity [89,118,162]. The activation of the MAPK1 signaling pathway was involved in cytokine production in SARS-CoV-2 [163]. Cadherin 1 (*CDH1*) is downregulated by both HG [164] and cathepsin L [165]. It was found that in cells infected by SARS-CoV-2, the expression of *CDH1* was significantly lowered [166].

Some data indicate the involvement of cathepsin L in the pathogenesis of diabetic kidney disease [167,168,169]. In proliferative diabetic retinopathy, the protein level of cathepsin L is significantly downregulated [150]. The comparative analysis of the CTSL-related gene network and networks of diabetic complications and metabolic abnormalities showed the presence of 47 CTSL-related genes/proteins in the CVD network, 14 genes/proteins in the diabetic neuropathy network, 47 in diabetic nephropathy, 30 in diabetic retinopathy, 83 in insulin resistance, and 7 in the beta-cell dysfunction network. All analyzed networks were enriched by CTSL-interacting genes/proteins with statistically significant *p*-values (less than 10^−30^, 10^−9^, 10^−24^, 10^−15^, 10^−39^, and 10^−4^, respectively).

According to the GO enrichment analysis, the identified genes are involved in the regulation of cell proliferation and migration, chemotaxis, gene expression, protein phosphorylation, the MAPK cascade, protein kinase B signaling, lipopolysaccharide-mediated signaling, protein import into the nucleus, and silencing by miRNA (Table 9). Other important processes include angiogenesis, the reactive oxygen species metabolic process, glucose homeostasis, acute-phase response, regulation of vascular endothelial growth factor production, apoptotic process, inflammatory response, and aging (Table S16).

#### 2.3.3. Intracellular Proteins Targeted by SARS-CoV-2

##### Network of Intracellular Proteins Targeted by SARS-CoV-2

**Figure 9:**
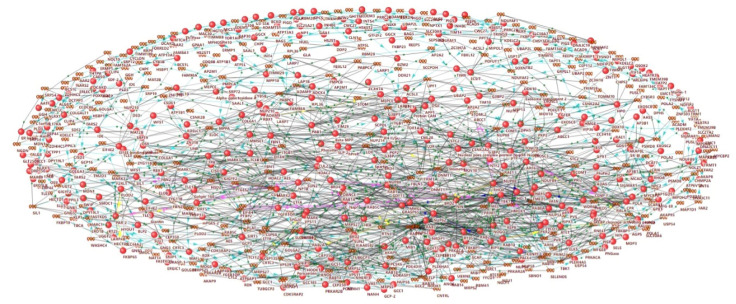
Gene network of human proteins targeted by SARS-CoV-2.

According to Gordon et al. [21], 332 human proteins are targeted by SARS-CoV-2. We reconstructed a gene network for these proteins (Figure 9) and found 1664 interactions within it (Table S17).

**Figure 10:**
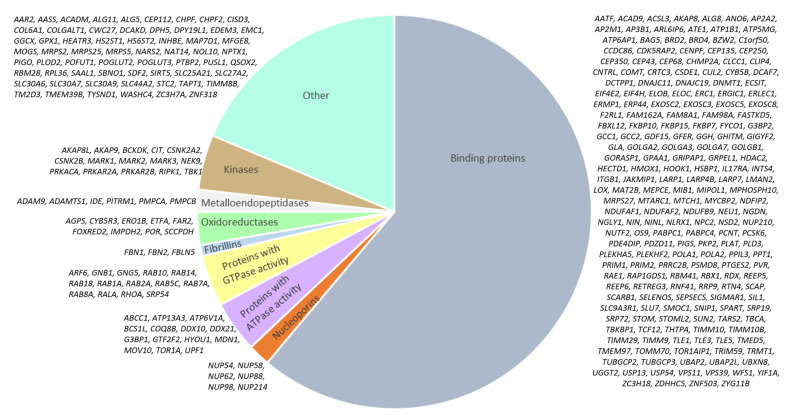
Molecules in the network of human proteins targeted by SARS-CoV-2.

Most of the nodes in the network (203 of 332) turned out to be proteins with binding activity. The network included the molecules that bind RNAs, macromolecular complexes, chaperones, enzymes, microtubules, guanosine triphosphate (GTP), and other molecules. There were some proteins with ATPase and GTPase activity, oxidoreductases, metalloendopeptidases, kinases, nucleoporins, and fibrillins (Figure 10).

As expected, viral process and intracellular transport were identified among enriched GO biological processes in which SARS-CoV-2-targeted proteins participate (Table 10). The list of overrepresented processes included protein transport, folding and heterotrimerization, protein targeting to mitochondrion, tRNA and mRNA transport, regulation of the mitotic cell cycle, regulation of cellular response to heat, and others. In addition, we found the regulation of glucose transport among the overrepresented processes.

We identified mov10 RISC complex RNA helicase (*MOV10*), Golgi reassembly stacking protein 1 (*GORASP1*), nucleoporin 62 (*NUP62*), cullin 2 (*CUL2*), golgin A2 (*GOLGA2*), OS9 endoplasmic reticulum lectin (*OS9*), Ras homolog family member A (*RHOA*), G3BP stress granule assembly factor 1 (*G3BP1*), RAB7A, member RAS oncogene family (*RAB7A*), and centrosomal protein 250 (*CEP250*) as the network components with the highest betweenness centrality values (Table S18). Among them, products of *G3BP1*, *MOV10*, *RAB7A*, and *RHOA* pose hydrolase activity; *CEP250* and *NUP62* regulate the protein localization to centrosomes; *GOLGA2* and *GORASP1* are associated with transport through the Golgi complex; and *CUL2* and *OS9* are involved in the protein ubiquitination.

The NADH: ubiquinone oxidoreductase complex assembly factor 1 (*NDUFAF1*), TM2 domain containing 3 (*TM2D3*), fatty acyl-CoA reductase 2 (*FAR2*), centrosomal protein 68 (*CEP68*), golgin A7 (*GOLGA7*), nucleolar protein 10 (*NOL10*), nucleoporin 58 (*NUP58*), centrosomal protein 112 (*CEP112*), nucleoporin 54 (*NUP54*), and quiescin sulfhydryl oxidase 2 (*QSOX2*) genes demonstrated the highest crosstalk specificity values (Table S19). The functions of the molecules encoded by these genes are quite diverse: nucleoporins are responsible for the transport of molecules across the nuclear envelope; fatty acyl-CoA reductase 2 is a fatty acid to fatty alcohols-converting enzyme; quiescin sulfhydryl oxidase 2 is an enzyme catalyzing the oxidation of sulfhydryl groups in peptide and protein thiols to disulfides with the reduction of oxygen to hydrogen peroxide; centrosomal proteins are components of the human centrosomes and are involved in cell division control; NADH: ubiquinone oxidoreductase complex assembly factor 1 is involved in the mitochondrial respiratory chain catalyzing the transfer of electrons from NADH to ubiquinone; TM2 domain-containing 3 regulates the signal cascades of cell death/proliferation; golgin A7 participates in the transport of proteins from the Golgi complex to the cell surface; and finally, nucleolar protein 10 is associated with late ribosomal RNA-processing events and the assembly of ribosomal particles [170].

Therefore, the key players of the network of SARS-CoV-2-targeted proteins are involved in the protein transport, ubiquitination and cleavage, biogenesis of ribosomes, response to reactive oxygen species and mitochondrial respiration and signal cascades of cell death/proliferation.

##### Comparative Analysis of the Network of Hyperglycemia and Network of Human Proteins Targeted by SARS-CoV-2

At the next step, we performed the mapping and comparative analysis of both reconstructed networks with assessment of network centralization, average number of neighbors, and network density. Among these parameters, the network centralization is a measure of how the nodes with high to low centrality are distributed. The centralization is higher if there are many clustered hubs in a network. The average number of neighbors reflects the overall connectivity of the nodes in a network. The network density describes the proportion of all possible links between nodes that are in fact observed in a network. A high network density measure shows the signal transduction effectiveness in a network [171].

It was revealed that molecules that make up the hyperglycemia-associated network are more tightly interconnected than those in the network of SARS-CoV-2-targeted proteins. The network centralization values were 0.61 and 0.046, average numbers of neighbors 84.764 and 4.322, and network density values 0.1 and 0.007, respectively. The obtained results indicate that the hyperglycemia-associated network represents close interactions between genes/proteins and it could be considered as a single module in the global human gene network. Oppositely, the participants of the network of SARS-CoV-2-targeted proteins seem to be not so tightly connected to each other. This is consistent with the potential of the virus to affect a huge number of cellular and physiological processes [172,173].

The intersection of the two analyzed networks revealed that eight genes (*DNMT1*, *FBN1*, *GDF15*, *GPX1*, *HMOX1*, *IDE*, *PLAT*, and *RHOA*) are common for them. The proteins encoded by these genes are very different in functional specialization.

DNA methyltransferase 1 encoded by *DNMT1* gene transfers methyl groups to DNA cytosine nucleotides that are responsible for maintaining DNA methylation patterns. Hyperglycemia increases the enzyme levels in retinal endothelial cells [174]. In turn, the changes in the retinal DNA methylation machinery induced by high glucose are involved in mitochondrial damage and persist after normoglycemia is restored, and therefore may be involved in the metabolic memory in diabetes [175]. It was shown that transient hyperglycemia directly upregulated *DNMT1* expression, leading to the hypermethylation of angiopoietin-1, long-lasting activation of NF-κB, and endothelial dysfunction [176]. In cultured SARS-CoV-2-infected lung epithelial cells, *DNMT1* was downregulated; however, this inhibition was not detected in COVID-19 patient’s lung tissues [177].

The fibrillin 1 gene (*FBN1*) encodes a preproprotein that further processes to fibrillin-1, an extracellular matrix glycoprotein, and a hormone asprosin. Fibrillin-1 is a structural component of calcium-binding microfibrils of the connective tissue. Asprosin, a fasting-induced glucogenic hormone, is secreted by white adipose tissue and is recruited to the liver, where it stimulates rapid glucose release into the circulation via the G protein-cAMP-PKA pathway. Humans and mice with insulin resistance show dramatically elevated plasma asprosin levels [178]. Under hyperglycemia conditions, the expression of *FBN1* is increased in the kidneys and decreased in the heart due to the epigenetic modifications [179]. A decrease in asprosin serum levels has been reported in patients with COVID-19 [180].

Growth differentiation factor 15 (GDF15) is a secreted ligand that binds to various transforming growth factor beta (TGF-β) receptors resulting in SMAD transcription factor activation. In addition to the signaling patterns of TGF-β, it also acts as a pleiotropic cytokine that participates in the response to cellular injury. Some data demonstrate that GDF-15 is involved in the regulation of inflammation, endothelial cell function, insulin sensitivity, and weight gain [181]. In COVID-19, GDF15 levels are associated with the disease severity and progression [182,183]. 

Glutathione peroxidase 1 (GPX1) is a selenium-dependent antioxidant enzyme essential for cell survival in oxidative stress. The *GPX1* expression is induced by hyperglycemia [184]. The increased GPX1 activity in hyperglycemic conditions could be adaptive and aimed at compensating a decrease in the enzyme protein level due to enhanced proteasome degradation [185]. Recent experimental data indicate the dual role of GPX1 in glucose and lipid metabolism: *GPX1* overexpression in the beta cells and insulin-responsive tissues lead to metabolic phenotypes similar to type 2 diabetes; meanwhile, *Gpx1−/−* mice develop insulin-dependent diabetes [186].

Heme oxygenase 1 (HO-1, *HMOX1*) is a key rate-limiting enzyme in the process of degradation of heme, the iron-containing molecule. HO-1 acts as antioxidant, anti-inflammatory, antiapoptotic and angiogenic factor through its by-products carbon monoxide (CO) and bilirubin, and can affect multiple cellular pathways involved in endothelial dysfunction and oxidative stress [187]. HO-1 demonstrates antiviral activity by interfering with the replication or activation of the interferon pathway [188]. It was shown that quercetin, a HO-1 inducer, reduced SARS-CoV-2 spike protein expression in kidney cell lines [189]. High glucose decreases *HMOX1* expression and protein activity in endothelial cells [190]. In turn, the induction of HO-1 alleviated oxidative and inflammatory response and endoplasmic reticulum stress induced by high glucose in cultured endothelial cells [191]. In different diabetic models, upregulating the HO system increases insulin secretion and reduces hyperglycemia. Similarly, CO also enhances insulin production and improves glucose metabolism [192].

Tissue-type plasminogen activator (tPA) encoded by the *PLAT* gene is a secreted serine protease that converts the proenzyme plasminogen to plasmin, a fibrinolytic enzyme. It is also involved in cell migration and tissue remodeling. The abnormal activity of the enzyme causes the disruptions in fibrinolysis, leading to excessive bleeding or thrombosis. The decreased activity of tPA could be a risk factor for type 2 diabetes [193]; its dysregulation can aggravate adverse cardiovascular events in hyperglycemia [194]. It was estimated that COVID-19 is associated with increased plasma thrombin generation [195]. Plasma tPA is elevated in COVID-19 patients. At the same time, the plasminogen activator inhibitor-1 (PAI-1) level is reduced [196] and is associated with increased mortality [197].

Insulin-degrading enzyme (IDE) is a zinc metallopeptidase that breaks down the intracellular insulin; it is also able to degrade glucagon, amylin, β-amyloid, and bradykinin. Moreover, IDE behaves as a heat shock protein and modulates the ubiquitin–proteasome system. Current data indicate that IDE acts as a regulator of insulin secretion and hepatic insulin sensitivity, and may participate in the crosstalk between the liver and beta cells. There is increasing evidence that improper IDE function, regulation, or trafficking might be involved in the pathogenesis of metabolic diseases [198,199].

Ras homolog family member A (RhoA, *RHOA*) is a small GTPase that regulates cell shape, attachment, and motility by promoting the reorganization of the actin cytoskeleton. RhoA participates in the regulation of smooth muscle tone and activates many downstream kinases. It was revealed that the RhoA/Rho-kinase pathway plays an important role in endothelial function and is implicated in cardiovascular disease, erectile dysfunction [200], and diabetic nephropathy [201]. Hyperglycemia causes the increase in *RHOA* expression in smooth muscles [202]. *RHOA* was identified among the hub genes playing a central role in COVID-19 immunopathogenesis [203].

Recently, Sardar et al. identified *HMOX1*, *DNMT1*, *PLAT*, *GDF1*, and *ITGB1* as hub genes that are involved in the host–virus interactions in SARS-CoV-2 infection [204]. According to our results, three of them (*HMOX1*, *DNMT1*, and *PLAT*) are common for the networks of hyperglycemia and SARS-CoV-2-targeted proteins. 

**Figure 11:**
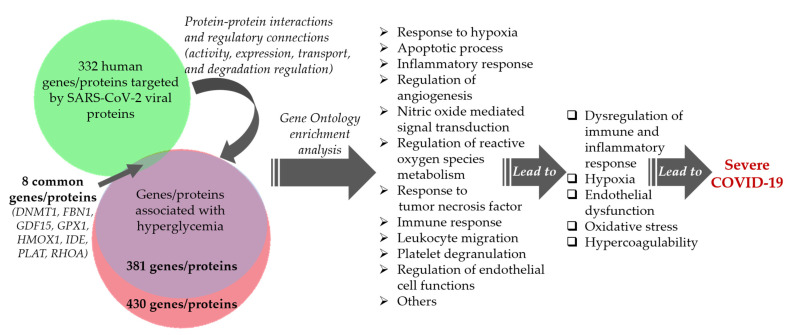
Interactions between hyperglycemia-associated network and network of SARS-CoV-2-targeted proteins contribute to severe COVID-19 in patients with diabetes.

We revealed, by the comparative analysis of two networks, that SARS-CoV-2-targeted proteins directly interact with 381 gene/proteins of the hyperglycemia network, i.e., almost all of them (Table S20). Among these interactions, there were a large number of protein–protein interactions, as well as regulatory relationships that concern the regulation of protein activity, expression, transport, and degradation. The GO enrichment analysis showed the involvement of these genes/proteins in the response to hypoxia, the apoptotic process, inflammatory response, regulation of angiogenesis, nitric oxide-mediated signal transduction, regulation of reactive oxygen species metabolism, response to tumor necrosis factor, immune response, leukocyte migration, platelet degranulation, regulation of endothelial cell functions, and others processes (Table S21). These findings are in consistence with the estimated pathophysiological abnormalities, such as hypercoagulability, endothelial dysfunction, oxidative stress, and dysregulation of the inflammatory and immune response, which characterize acute and long COVID-19 (Figure 11).

##### Comparative Analysis of the Networks of Diabetic Complications and Network of Human Proteins Targeted by SARS-CoV-2

**Figure 12:**
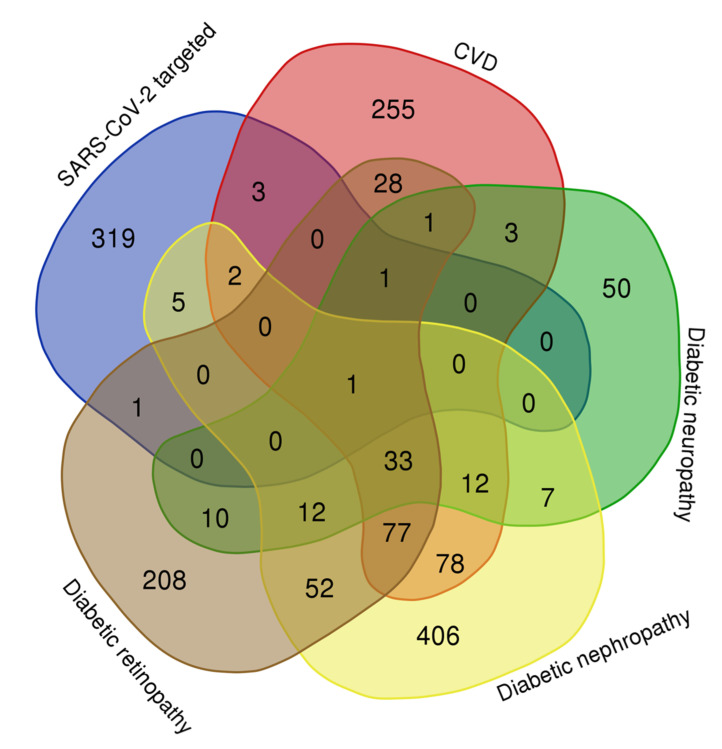
Venn diagram of intersection of the lists of SARS-CoV-2-targeted proteins and gene/protein sets associated with cardiovascular disease, diabetic neuropathy, diabetic nephropathy, and diabetic retinopathy. Numbers show the gene/protein count.

In this work, we revealed some intersections between genes/proteins associated with diabetes complications and those of SARS-CoV-2-targeted proteins (Figure 12). Seven molecules were found when assessing the CVD network (*COMT*, *GDF15*, *GPX1*, *HMOX1*, *LOX*, *PLAT*, and *SELENOS*), eight in the network of diabetic nephropathy (*DNMT1*, *F2RL1*, *GDF15*, *HDAC2*, *HMOX1*, *HYOU1*, *LOX*, and *RHOA*), three in the network of diabetic retinopathy (*HMOX1*, *NUTF2*, and *PLAT*), and two in the diabetic neuropathy network (*HMOX1* and *PLAT*).

The role of *DNMT1*, *GDF15*, *GPX1*, *HMOX1*, *PLAT*, and *RHOA* were considered in the previous section. We identified *HMOX1* as a shared hub for all analyzed networks. This corresponds to the broad biological functions of HO-1. The beneficial effects of HO-1 and its reaction products in diabetic vascular complications include anti-inflammatory, antiproliferative, antiapoptotic, and immunomodulatory activity [205]. It was revealed that polymorphism in the *HMOX1* promoter is associated with CVD in subjects with diabetes [206]. The serum levels of HO-1 are reduced in patients with diabetic retinopathy [207]. In mice, HO-1 deficiency contributes to diabetic kidney disease [208]. Accordingly, the induction of the enzyme demonstrated a protective effect in diabetic nephropathy [209]. HO-1 mitigates cytokine storm and lung injury in mouse models of sepsis and may exert antivirus activity [210]. Therefore, it could be speculated that the suppression of HO-1 in hyperglycemia is a promoting mechanism for diabetic complications and a more severe COVID-19 course.

The *PLAT* gene was found in the networks of hyperglycemia, CVD, diabetic neuropathy, and retinopathy. This is consistent with data on the important role of fibrinolysis disorders in the development of diabetic vascular complications [211] and COVID-19 [212].

We found *GDF15* in the networks of CVD and diabetic nephropathy. In type 2 diabetes, GDF15 is associated with both macrovascular and microvascular complications [213,214]. At the same time, GDF-15 is considered an indicator of COVID-19 severity [182,183].

The lysyl oxidase gene (*LOX*) was also revealed in the networks of CVD and diabetic nephropathy. The enzyme is involved in the crosslinking of collagens and elastin, and is supposed to be involved in the impairment of the elastic component of lungs in COVID-19 [215]. The LOX gene polymorphisms are associated with CVD [216] and it was proposed as a drug target for CVD therapy [217]. An enhanced *LOX* expression in the kidneys was found in rats with diabetic nephropathy [218].

Selenoprotein S (*SELENOS*), which was found in the network of CVD, is a transmembrane protein involved in the degradation of misfolded proteins in the endoplasmic reticulum; it is involved in inflammation, oxidative stress, endoplasmic reticulum stress, and glucose metabolism [219]. Selenoprotein S is highly expressed in the blood vessels [220] and is supposed to be a target in diabetic macroangiopathy [219].

Three more genes (*F2RL1*, *HDAC2*, and *HYOU1*) identified in the diabetic nephropathy network deserve to be mentioned. F2R-like trypsin receptor 1, or protease-activated receptor 2 (*F2RL1*), is a G-protein coupled receptor; it stimulates vascular smooth muscle relaxation, the dilation of blood vessels, and increases blood flow; it is also involved in the inflammatory and immune response. F2R-like trypsin receptor 1 was shown to aggravate diabetic nephropathy progression [221]. SARS-CoV-2 viral protein ORF9c directly interacts with PAR2 with F2R-like trypsin receptor 1; moreover, it was speculated that the activation of protease-activated receptors by proteases plays a role in COVID-19-induced hyperinflammation [222]. Histone Deacetylase 2 (*HDAC2*) determines the acetylation status of histones and plays an important role in diabetic nephropathy via the excessive accumulation of the extracellular matrix in the kidneys and epithelial-to-mesenchymal transition of renal tubular epithelial cells [223,224]. Hypoxia Upregulated 1 (*HYOU1*) is a heat shock protein accumulated in the endoplasmic reticulum under hypoxic conditions, which is important for protein folding and secretion in the endoplasmic reticulum and is associated with apoptosis. In patients with diabetic nephropathy, HYOU1 was upregulated in tubular epithelial cells [225].

Nuclear transport factor 2 (*NUTF2*), identified in the diabetic retinopathy network, is a cytosolic factor that facilitates the transport of the proteins into the nucleus. The level of the factor was lower in patients with diabetic retinopathy; its overexpression showed a protective effect against diabetic retinopathy [226].

The participants of the gene networks of diabetic complications were directly linked to some SARS-CoV-2-targeted proteins (Table S22). It turned out that SARS-CoV-2-targeted proteins had interactions with 415, 583, 339, and 110 genes/proteins in the network of CVD, diabetic nephropathy, diabetic retinopathy, and diabetic neuropathy, respectively. The most overrepresented GO biological processes for these gene sets were cytokine-mediated signaling, response to hypoxia, inflammatory response, regulation of blood pressure and angiogenesis, regulation of cell proliferation, migration and apoptosis, as well as protein kinase B, ERK1 and ERK2, and phosphatidylinositol 3-kinase signaling (Table 11 and Table S23).

##### Comparative Analysis of the Networks of Insulin Resistance, Beta-Cell Dysfunction, and Human Proteins Targeted by SARS-CoV-2

The gene network associated with insulin resistance contained 1452 genes/proteins (Table S6). The intersection of this network with the network of human proteins targeted by SARS-CoV-2 included 13 genes/proteins: *HMOX1*, *PLAT*, *GDF15*, *DNMT1*, *F2RL1*, *GPX1*, *SELENOS*, *IDE*, *BRD2*, *ERP44*, *PCNT*, *RAB10*, and *SCARB1*. Among them, *HMOX1*, *PLAT*, *GDF15*, *DNMT1*, *F2RL1*, *GPX1*, *SELENOS*, and *IDE* were also involved in the networks of diabetic complications and hyperglycemia.

It was found that the induction or overexpression of *HMOX1* improves the insulin sensitivity and glucose tolerance [227,228]. The elevated levels of PLAT and GDF15 were associated with insulin resistance [229,230,231]. The inverse correlation of *DNMT1* expression with insulin sensitivity was observed in adipose tissue [232]. The lack of F2rl1 in mice was associated with the protection from the insulin resistance induced by a high-fat diet [233]. The overexpression of GPX1 was shown to cause insulin resistance [234,235]. The expression of *SELENOS* and a number of SNPs in it were associated with the homeostasis model assessment of insulin resistance [219,236]. The IDF inhibition improves insulin sensitivity [237] and the upregulation of IDE could be used as a treatment for insulin resistance [238].

Bromodomain-containing 2 (*BRD2*) is a transcriptional regulator that participates in mitosis. BRD2 can induce insulin resistance through the mTOR/Akt signaling pathway and an inflammatory response in adipose tissue [239]. Endoplasmic Reticulum Protein 44 (*ERP44*) is a pH-regulated chaperone and could participate in protein quality control at the endoplasmic reticulum–Golgi interface. The decreased cellular level of ERP44 is associated with insulin resistance [240]. Pericentrin (*PCNT*) is an integral component of the pericentriolar material and is involved in the functioning of the centrosomes, cytoskeleton, and cell-cycle progression. Mutations in *PCNT* are associated with severe insulin resistance and diabetes [241]. RAB10 is a member of the RAS oncogene family and a small GTPase that regulates intracellular vesicle trafficking. The adipose RAB10 is involved in systemic insulin sensitivity, as RAB10 is required for insulin-stimulated GLUT4 translocation to the plasma membrane that is responsible for glucose uptake [242]. Scavenger receptor class B member 1 (*SCARB1*) is a high-density lipoprotein cholesterol plasma membrane receptor and the polymorphisms in this gene are associated with insulin resistance [243,244].

In the gene network associated with insulin resistance, there were 1163 genes/proteins directly linked with participants of the network of proteins targeted by SARS-CoV-2 (Table S24). The GO enrichment analysis showed that these genes are involved in the cytokine-mediated signaling pathway, apoptotic process, response to inflammation and hypoxia, and other processes (Table S24).

The gene network associated with beta-cell dysfunction included 72 genes/proteins (Table S7). Fifty-four of them demonstrated interactions with the proteins targeted by SARS-CoV-2 (Table S25). These 54 genes/proteins are involved in the GO biological processes related to the cytokine-mediated signaling pathway, apoptotic process, release of cytochrome c from mitochondria, T cell homeostasis, cell proliferation, and others (Table S25). Among the identified genes, *TNF* and *CASP3* were associated with COVID-19-related networks [92,127].

### 2.4. Discussion

The results of our study indicate that in patients with diabetes, SARS-CoV-2 triggers a cascade of molecular events that can be considered in terms of molecular networks with a number of positive and negative feedback loops, bypasses, and parallel regulatory pathways. In diabetes, HG induces a wide range of changes in the gene expression, forming a pathophysiological basis for an inappropriate response to stressors including SARS-CoV-2. According to our data, the hyperglycemia-related network includes 430 genes/proteins that are involved in the inflammatory pathways, response to hypoxia, regulation of cell proliferation, angiogenesis, apoptosis, and other processes. The virus can induce further disturbances in the biochemical and pathophysiological processes induced by hyperglycemia.

We have shown that the networks of SARS-CoV-2 entry-supporting proteins (ACE2, DPP4, CTSB and CTSL) are significantly enriched with the genes/proteins associated with hyperglycemia. In addition, the molecules forming the networks of human proteins related to SARS-CoV-2 were found to be significantly overrepresented in the gene networks of the diabetes complications (CVD, diabetic neuropathy, diabetic nephropathy, and diabetic retinopathy), as well as in the insulin resistance and beta-cell dysfunction networks. These findings are consistent with clinical data on more severe courses and poorer outcomes of COVID-19 in subjects with diabetes [2,3] and give further support to notion of parallels between COVID-19 and diabetes pathology [8].

The clinical evidence supports the role of some molecules revealed in this work in COVID-19 pathogenesis: ANGPT2 [48,49], CCL2 [50], ICAM1 [52,53], VCAM-1 [52], MIR21 [54], MMP9 [55,56], STAT3 [57], HMGB1 [58,59], SIRT1 [62,63], AGTR1 [66], APOE4 [69,70], ACE [73], CCL11 [86,87], FGF2 [89], TNF [92], PPAR-γ [95], CASP8 [115], IL8 [117], IL6 [118], PRL [119], SP1 [122], TLR4 [123], BDNF [124], CASP1 [125], CASP3 [126,127], IL18 [128], IL10 [129], TGFB1 [130], ANXA2 [131,132], HPSE [162], MAPK1 [163], CDH1 [166], FBN1 [180], GDF15 [182,183], PLAT [195,196,197], RHOA [203], HMOX1 [204], LOX [215], and others.

According to the GO enrichment analysis, the molecules associated with the proteins related to SARS-CoV-2 are involved in the immune and inflammatory response, acute-phase response, interleukin-8 production, oxidative stress, regulation of cytokine production, response to hypoxia, regulation of vascular endothelial cell proliferation, glucose homeostasis, fibrinolysis, extracellular matrix formation, tissue remodeling, apoptosis, regulation of cell proliferation and migration, angiogenesis, aging, gene expression, phosphatidylinositol 3-kinase signaling, protein kinase B signaling, DNA methylation, and protein phosphorylation. These processes could provide a pathophysiological basis for a more severe clinical course of COVID-19 in subjects with diabetes [172,173].

### 2.5. Study Limitations

Our study is not without limitations. The gene network reconstruction was based on the text-mining of PubMed/Medline-indexed publications only. Therefore, we cannot exclude that some relevant information has been missed or some of the revealed interactions are false-positive. The study is a hypothesis-generating one. The role of some identified genes/proteins as mediators of a more severe clinical course and worse outcomes of COVID-19 in patients with diabetes needs further experimental verification.

## 3. Materials and Methods

**Figure 13:**
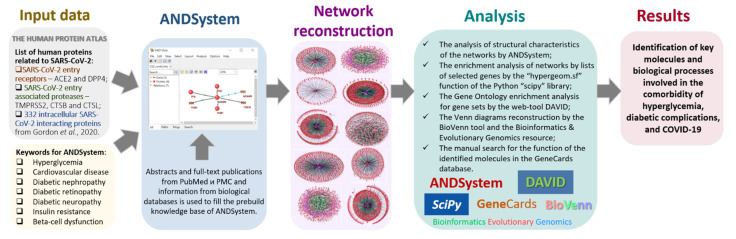
Flowchart of the study.

The study design is presented as a flowchart in Figure 13.

The gene networks were automatically reconstructed by the ANDSystem [23,24], version: 22.0118b686_2022 (ICG SB RAS, Novosibirsk, Russia), available online at http://www-bionet.sscc.ru/and/cell/ (accessed on 10 January 2022).

The structural characteristics of the studied networks were analyzed by the ANDSystem function “Statistics” of the “Analysis” section. It was used to find the betweenness centrality coefficients, the network centralization, the average number of neighbors, the network density, and the CTS values. The CTS reflects the degree to which a particular node is specifically involved in the studied network. CTS is calculated as following: CTS = K_i_/M_i_, where K_i_ stands for the number of interactions of a particular i-th gene in the analyzed gene network and M_i_ stands for the number of interactions of this i-th gene in the global human gene network of the ANDSystem [24,26].

The enrichment of the analyzed gene networks by lists of selected genes was assessed according to the hypergeometric distribution by the “hypergeom.sf” function of the “scipy” library of the Python programming language [245].

The GO enrichment analysis for gene sets was performed by the web-tool DAVID, version 6.8 (LHRI, Frederick, MD, USA) [45]. It is available online: https://david.ncifcrf.gov/home.jsp (accessed on 20 February 2022). The used parameters were: organism, “Homo sapiens”; Gene_Ontology, “GOTERM_BP_DIRECT”; the cut-off for the statistical significance was set as *p*-values with FDR correction lower than 0.05.

The information on the function of the identified genes was obtained from the database GeneCards [170]. It is available online at https://www.genecards.org/ (accessed on 25 February 2022).

The Venn diagram demonstrating the interactions of genes from the gene networks (Figure 11) was made by the BioVenn web application (available at https://www.biovenn.nl/index.php, accessed on 18 February 2022). The Venn diagram showing the interactions of the gene lists associated with the diabetes complications and the proteins targeted by SARS-CoV-2 (Figure 12) was made by the “Bioinformatics & Evolutionary Genomics” resource (available online at http://bioinformatics.psb.ugent.be/webtools/Venn/, accessed on 19 February 2022).

## 4. Conclusions

In this work, we have demonstrated, for the first time, that the hyperglycemia network and the networks of SARS-CoV-2-targeted proteins have a number of paths that interact with each other. We revealed that SARS-CoV-2-targeted proteins directly regulate physical interactions with 381 gene/proteins of the hyperglycemia network, i.e., almost all of them. The proteins associated with hyperglycemia and targeted by SARS-CoV-2 proteins are involved in glucose homeostasis, fibrinolysis, extracellular matrix formation, cell migration, tissue remodeling, DNA methylation, response to cellular injury, hypoxia, immune response, inflammation, and oxidative stress. We identified *HMOX1* as a shared hub for all analyzed networks. The *PLAT* gene could be a possible hub that links hyperglycemia, COVID-19, and negative cardiovascular events. Most elements of the hyperglycemia-associated network demonstrate protein–protein or regulatory links with the SARS-CoV-2-targeted proteins. The involvement of these interactions in the cytokine network, inflammation and immunity, angiogenesis and response to hypoxia, oxidative stress, apoptosis, and endothelial cell functions seems to form a pathogenic basis for a more a severe course of COVID-19 in subjects with diabetes.

A number of genes/proteins targeted by SARS-CoV-2 (ACE2, BRD2, COMT, CTSB, CTSL, DNMT1, DPP4, ERP44, F2RL1, GDF15, GPX1, HDAC2, HMOX1, HYOU1, IDE, LOX, NUTF2, PCNT, PLAT, RAB10, RHOA, SCARB1, and SELENOS) were found in the networks of vascular diabetic complications and insulin resistance. According to the GO enrichment analysis, the identified molecules are involved in cytokine-mediated signaling, response to hypoxia, inflammatory response, regulation of blood pressure and angiogenesis, regulation of cell proliferation, migration and apoptosis, as well as protein kinase B, ERK1 and ERK2, and phosphatidylinositol 3-kinase signaling, and other processes.

The results obtained contribute to the deeper understanding of the molecular pathophysiology of COVID-19-induced disorders in subjects with diabetes. The functional significance of the identified hub molecules and their potential value as therapeutic targets requires further research.

## Figures and Tables

**Table 1:** Top 10 GO biological processes overrepresented for hyperglycemia-associated genes found by DAVID (*p*-values with FDR correction < 0.05).

Gene Ontology Biological Process	Gene Ontology ID	Genes	*p*-Values with FDR Correction
Glucose homeostasis	GO:0042593	*ADIPOQ*, *ADIPOR1*, *ADRA2A*, *CEBPA*, *CNR1*, *FBN1*, *G6PC1*, *GCGR*, *GCK*, *HIF1A*, *HNF1A*, *IL6*, *INS*, *IRS1*, *LEP*, *LEPR*, *MTNR1B*, *NEUROD1*, *NGFR*, *PAX6*, *PDK4*, *PDX1*, *POMC*, *PPARG*, *PRKAA1*, *PRKAA2*, *RBP4*, *SIRT6*, *SLC2A4*, *STAT3*, *STK11*, *TCF7L2*	7.92 × 10^−23^
Inflammatory response	GO:0006954	*AGER*, *AOC3*, *CALCA*, *CCL11*, *CCL2*, *CD40LG*, *CRH*, *CRP*, *CXCL12*, *CXCL8*, *CXCR4*, *CYBB*, *ECM1*, *F2R*, *FOS*, *FPR1*, *HMGB1*, *IL10*, *IL13*, *IL18*, *IL19*, *IL1A*, *IL1B*, *IL22*, *IL6*, *NFATC3*, *NFE2L2*, *NGFR*, *NLRP3*, *NOX4*, *PIK3CD*, *PIK3CG*, *PRKD1*, *PTGER2*, *PTGS2*, *RAC1*, *RELA*, *SELE*, *SELP*, *SPP1*, *TBXA2R*, *TGFB1*, *THBS1*, *TLR2*, *TLR4*, *TNF*, *TNFRSF11B*	6.69 × 10^−17^
Response to hypoxia	GO:0001666	*ADIPOQ*, *ADM*, *AGER*, *ANGPT2*, *ANGPTL4*, *CASP1*, *CASP3*, *CAT*, *CAV1*, *CCL2*, *CDKN1B*, *CREB1*, *CXCL12*, *CXCR4*, *DPP4*, *EGR1*, *EPO*, *HIF1A*, *HMOX1*, *HSPD1*, *LEP*, *MB*, *MMP2*, *NOS1*, *NOS2*, *NOX4*, *PLAT*, *PLAU*, *PPARA*, *PRKAA1*, *PRKCB*, *RYR2*, *SOD2*, *TGFB1*, *THBS1*, *TLR2*, *UCP2*, *VCAM1*, *VEGFA*, *VEGFB*	1.41 × 10^−23^
Positive regulation of angiogenesis	GO:0045766	*ADM*, *ANGPT2*, *ANGPTL4*, *CCL11*, *CX3CL1*, *CXCL8*, *CYBB*, *DDAH1*, *ECM1*, *F3*, *FGF2*, *GATA4*, *HGF*, *HIF1A*, *HMOX1*, *IL1A*, *IL1B*, *KDR*, *NFE2L2*, *NOS3*, *PRKCB*, *PRKD1*, *PTGIS*, *SERPINE1*, *SIRT1*, *TBXA2R*, *THBS1*, *VEGFA*	9.5 × 10^−17^
Positive regulation of cell proliferation	GO:0008284	*ADM*, *ADRA2A*, *AR*, *ATF3*, *AVP*, *AVPR1A*, *BCL2*, *CCK*, *CCN2*, *CCND2*, *CD47*, *CDK2*, *CDKN1B*, *CRH*, *CTF1*, *DPP4*, *EDN1*, *EGFR*, *EIF5A*, *EPO*, *ESM1*, *F2R*, *FABP1*, *FGF2*, *FGF21*, *FN1*, *GDNF*, *GHRH*, *HGF*, *IFNG*, *IGF1*, *IGF1R*, *IGF2*, *IL2*, *IL6*, *INS*, *IRS1*, *IRS2*, *KDR*, *LEP*, *MAPK1*, *MYC*, *NAMPT*, *NOTCH1*, *NRG1*, *NTN1*, *PDX1*, *PRKAA1*, *PTEN*, *REG1A*, *RELA*, *S100B*, *SIRT1*, *STAT3*, *TGFB1*, *THBS1*, *VEGFA*, *VIP*	2.86 × 10^−21^
Negative regulation of apoptotic process	GO:0043066	*ALB*, *ANGPT1*, *ANGPTL4*, *AVP*, *BCL2*, *CASP3*, *CAT*, *CCND2*, *CD40LG*, *CD44*, *CDKN1A*, *CDKN1B*, *DDAH2*, *EGFR*, *FABP1*, *FOXO1*, *GAS6*, *GCG*, *GDNF*, *GLO1*, *GSK3B*, *HSPD1*, *IGF1*, *IGF1R*, *IL10*, *IL2*, *IL4*, *IL6*, *KDR*, *LEP*, *LTF*, *MAPK7*, *MMP9*, *MPZ*, *MYC*, *NGF*, *NGFR*, *NQO1*, *PAX4*, *PIK3R1*, *PRKAA1*, *PRKAA2*, *PRNP*, *PTEN*, *RELA*, *SIRT1*, *SNCA*, *SOCS3*, *SOD2*, *STAT3*, *THBS1*, *TP53*, *UCP2*, *VEGFA*, *VEGFB*	1.69 × 10^−19^
Positive regulation of protein kinase B signaling	GO:0051897	*ANGPT1*, *CD28*, *EGFR*, *F3*, *FGF2*, *GAS6*, *GPX1*, *HPSE*, *IGF2*, *IL18*, *IL6*, *INS*, *LEP*, *MTOR*, *NOX4*, *NRG1*, *PIK3CG*, *RICTOR*, *TCF7L2*, *TGFB1*, *THBS1*, *TNF*, *TXN*, *VEGFB*	8.49 × 10^−16^
Positive regulation of transcription from RNA polymerase II promoter	GO:0045944	*APP*, *AR*, *ARNTL*, *ATF3*, *CD28*, *CEBPA*, *CREB1*, *CREM*, *CTNNB1*, *DCN*, *DDIT3*, *EDN1*, *EGFR*, *EGR1*, *FGF2*, *FOS*, *FOXO1*, *FOXO3*, *GALR1*, *GATA4*, *GDNF*, *GSK3B*, *HGF*, *HIF1A*, *HMGA1*, *HMGB1*, *HNF1A*, *IFNG*, *IGF1*, *IL10*, *IL18*, *IL1A*, *IL1B*, *IL2*, *IL4*, *IL6*, *JUN*, *MAFA*, *MAFB*, *MAPK7*, *MEF2A*, *MEF2C*, *MEN1*, *MYC*, *NAMPT*, *NCK1*, *NEUROD1*, *NEUROG3*, *NFAT5*, *NFATC3*, *NFE2L2*, *NLRP3*, *NOS1*, *NOTCH1*, *NR1H2*, *NRG1*, *OGT*, *PARP1*, *PAX3*, *PAX6*, *PDX1*, *PIK3R1*, *POMC*, *PPARA*, *PPARG*, *PRKD1*, *PTH*, *RELA*, *SERPINE1*, *SIRT1*, *SIRT2*, *SP1*, *SREBF1*, *STAT3*, *TCF7L2*, *TGFB1*, *TLR2*, *TLR4*, *TNF*, *TP53*, *VEGFA*	2.25 × 10^−19^
Aging	GO:0007568	*ADM*, *ADRB3*, *AGT*, *ARG1*, *CALCA*, *CAT*, *CCL2*, *CCN2*, *CNR1*, *COL3A1*, *CREB1*, *DCN*, *EPO*, *FGF2*, *FOS*, *FOXO3*, *IGFBP1*, *IL10*, *IL6*, *JUN*, *KL*, *NFE2L2*, *NQO1*, *PTEN*, *RELA*, *RETN*, *SERPINF1*, *SNCA*, *SOD1*, *SREBF1*, *STAT3*, *TGFB1*, *UCP2*, *VCAM1*	2.47 × 10^−18^
Response to drug	GO:0042493	*ABCA1*, *ABCC8*, *APOA1*, *ARG1*, *BCL2*, *BGLAP*, *CASP3*, *CAT*, *CCND1*, *CDH1*, *CDH3*, *CDKN1A*, *CDKN1B*, *CREB1*, *CRH*, *CTNNB1*, *CYBB*, *DUSP6*, *FOS*, *GATA4*, *GIP*, *HSPD1*, *ICAM1*, *IFNG*, *IL10*, *IL4*, *IL6*, *JUN*, *KCNJ11*, *LCN2*, *LPL*, *MYC*, *NEUROD1*, *PAX4*, *PDX1*, *PPARG*, *PTEN*, *PTGS2*, *PTH*, *RELA*, *SMPD1*, *SNCA*, *SOD1*, *SOD2*, *SORD*, *SREBF1*, *SST*, *STAT3*, *TBXA2R*, *TGFB1*, *THBS1*, *TIMP4*, *TNFRSF11B*, *TXNIP*, *VEGFB*	1.44 × 10^−27^

**Table 2:** Types of associations of the genes/proteins from hyperglycemia-related and ACE2-related networks with high glucose (HG) and ACE2.

	HG	Gene Expression is Upregulated by HG	Gene Expression is Downregulated by HG	Molecules with Hyperglycemic Activity	Molecules with Antihyperglycemic Effect	Other Relations
ACE2						
**Molecules are upregulated by ACE2**		*BCL2*, *CCND1*, *MMP2*, *NOS1*, *NOS3*, *SOD1*, *UCP2*	*BCL2*, *CDH1*, *NOS3*	*IL1B*, *NOS2*		*NPHS1*, *SIRT6*
**Molecules are downregulated by ACE2**		*ANGPT2*, *CCL2*, *CCN2*, *HMGB1*, *ICAM1*, *MIR21*, *MMP9*, *STAT3*, *VCAM1*	*VEGFA*	*AGTR1*	*STAT3*	*ACE*, *ICAM1*
**Molecules upregulating ACE2**		*HMGB1*			*INS*	
**Molecules downregulating ACE2**		*EDN1*	*SIRT1*	*AGTR1*	*INS*, *SIRT1*	*ACE*, *ALB*, *APOE*
**Other relations**		*AGT*	*CAT*, *IRS1*	*GCG*		*CALM1*

**Table 3:** The most overrepresented GO biological processes that are common for the sets of genes linked with ACE2 and associated with hyperglycemia, CVD, diabetic neuropathy, diabetic nephropathy, diabetic retinopathy, and insulin resistance.

Gene Ontology Biological Process	Gene Ontology ID	*p*-Values with FDR Correction					
		Hyperglycemia	CVD	Diabetic Neuropathy	Diabetic Nephropathy	Diabetic Retinopathy	Insulin Resistance
Positive regulation of cell migration	GO:0030335	3.28 × 10^−5^	9.10 × 10^−4^	1.11 × 10^−5^	3.60 × 10^−5^	7.24 × 10^−6^	3.46 × 10^−4^
Negative regulation of gene expression	GO:0010629	6.51 × 10^−7^	2.68 × 10^−7^	0.0155	6.72 × 10^−9^	1.93 × 10^−8^	6.51 × 10^−7^
Positive regulation of vascular smooth muscle cell proliferation	GO:1904707	1.28 × 10^−4^	3.12 × 10^−4^	0.0155	4.46 × 10^−4^	2.53 × 10^−4^	0.0213
Positive regulation of phosphatidylinositol 3-kinase signaling	GO:0014068	3.28 × 10^−5^	0.0121	0.0277	1.62 × 10^−4^	8.03 × 10^−5^	8.51 × 10^−4^
Positive regulation of cell proliferation	GO:0008284	1.45 × 10^−4^	1.68 × 10^−4^	0.0494	4.29 × 10^−4^	0.0032	5.49 × 10^−5^
Negative regulation of apoptotic process	GO:0043066	1.32 × 10^−4^	0.0028	0.0489	6.47 × 10^−5^	0.0122	4.01 × 10^−5^
Response to hypoxia	GO:0001666	5.05 × 10^−7^	8.51 × 10^−7^		1.20 × 10^−9^	6.91 × 10^−7^	4.30 × 10^−8^
Response to lipopolysaccharide	GO:0032496	3.28 × 10^−5^	3.43 × 10^−7^		4.43 × 10^−9^	3.81 × 10^−7^	1.82 × 10^−8^
Nitric oxide mediated signal transduction	GO:0007263	6.11 × 10^−6^	1.63 × 10^−7^		2.08 × 10^−5^	3.63 × 10^−4^	1.85 × 10^−6^
Positive regulation of vascular endothelial cell proliferation	GO:1905564	4.15 × 10^−4^	3.52 × 10^−5^		1.48 × 10^−6^	2.49 × 10^−5^	1.70 × 10^−4^

**Table 4:** Types of associations of the genes/proteins from hyperglycemia-related and DPP4-related networks with high glucose (HG) and DPP4.

	HG	Gene Expression Is Upregulated by HG	Gene Expression Is Downregulated by HG	Molecules with Hyperglycemic Activity	Molecules with Antihyperglycemic Effect	Other Relations
DPP4						
**Genes are upregulated by DPP4**		*CD36*, *CD8A*, *CRP*, *IL6*, *MMP2*, *SPP1*	*CD44*, *HIF1A*, *VEGFA*		*PPARG*	*PLAT*
**Genes are downregulated by DPP4**		*CCL11*, *FGF2*, *HMGB1*, *MMP9*, *NPY*, *THBS1*, *VIP*	*PPY*	*CREB1*, *EGR1*, *GCG*, *GIP*	*ADIPOQ*, *EPO*, *GLP1R*, *INS*, *SERPINF1*	*CXCL12*, *GHRH*, *NPHS1*
**Molecules that upregulate *DPP4***		*CCL11*, *EGFR*, *IFNG*, *TNF*			*INS*	*IL2*, *IL13*
**Molecules that downregulate *DPP4***		*NPY*, *TLR4*			*MYC*	*PTH*, *TFPI*
**Other relations**		*CXCR4*, *FN1*	*CDH1*	*GCG*, *NOS2*	*CAV1*, *KL*	*HNF1A*, *LGALS3*

**Table 5:** Most overrepresented GO biological processes that are common for the sets of genes linked with DPP4 and are associated with hyperglycemia, CVD, diabetic neuropathy, diabetic nephropathy, diabetic retinopathy, insulin resistance, and beta-cell dysfunction.

Gene Ontology Biological Process	Gene Ontology ID	*p*-Values with FDR Correction						
		Hyperglycemia	CVD	Diabetic Neuropathy	Diabetic Nephropathy	Diabetic Retinopathy	Insulin Resistance	Beta-Cell Dysfunction
Response to hypoxia	GO:0001666	3.68 × 10^−13^	2.00 × 10^−7^	1.50 × 10^−5^	8.14 × 10^−13^	1.93 × 10^−9^	3.71 × 10^−10^	
Positive regulation of ERK1 and ERK2 cascade	GO:0070374	1.78 × 10^−9^	7.39 × 10^−5^	3.06 × 10^−6^	2.13 × 10^−10^	1.14 × 10^−8^	1.10 × 10^−10^	
Positive regulation of smooth muscle cell proliferation	GO:0048661	1.78 × 10^−9^	2.23 × 10^−4^	1.55 × 10^−4^	1.47 × 10^−6^	2.11 × 10^−7^	2.41 × 10^−4^	
Response to activity	GO:0014823	1.28 × 10^−8^	8.42 × 10^−5^	1.03 × 10^−4^	7.24 × 10^−6^	5.26 × 10^−4^	9.71 × 10^−4^	
Positive regulation of interleukin-8 production	GO:0032757	3.34 × 10^−5^	9.36 × 10^−6^	0.0022	4.08 × 10^−8^	1.48 × 10^−7^	1.20 × 10^−6^	
Aging	GO:0007568	2.59 × 10^−4^	0.0016	1.94 × 10^−4^	5.18 × 10^−5^	5.14 × 10^−6^	1.87 × 10^−4^	
Positive regulation of phosphatidylinositol 3-kinase signaling	GO:0014068	0.0012	4.54 × 10^−4^	1.94 × 10^−4^	1.09 × 10^−8^	5.43 × 10^−7^	5.45 × 10^−4^	3.07 × 10^−4^
Negative regulation of lipid storage	GO:0010888	8.41 × 10^−5^	5.31 × 10^−6^	0.0022	4.58 × 10^−6^	1.71 × 10^−6^	2.52 × 10^−5^	
Cellular response to lipopolysaccharide	GO:0071222	2.59 × 10^−4^	2.00 × 10^−7^	0.0022	7.19 × 10^−7^	5.14 × 10^−6^	6.64 × 10^−8^	
Acute-phase response	GO:0006953	1.51 × 10^−4^	6.18 × 10^−4^	8.12 × 10^−4^	4.08 × 10^−5^	8.33 × 10^−6^	0.0039	

**Table 6:** Types of associations of the genes/proteins from hyperglycemia-related and cathepsin B (CTSB)-related networks with high glucose (HG) and cathepsin B (*STSB*).

	HG	Gene Expression Is Upregulated by HG	Gene Expression Is Downregulated by HG	Molecules with Hyperglycemic Activity	Molecules with Antihyperglycemic Effect	Other Relations
*CTSB*						
**Genes are upregulated by cathepsin B**		*BAX*, *BCL2*, *BDNF*, *CASP1*, *CASP3*, *CASP8*, *CCL2*, *CXCL8*, *DCX*, *IL18*, *MMP9*, *MTOR*, *NLRP3*, *PRL*, *PTEN*	*BCL2*, *CCK*, *VEGFA*	*PRL*	*IL4*, *IL18*, *MTOR*	*APOE*, *HSPG2*
**Genes are downregulated by cathepsin B**		*APP*, *FN1*	*BGLAP*, *SIRT1*	*CDKN1B*	*SIRT1*	
**Molecules that upregulate *CTSB***		*CASP8*, *CXCL8*, *IL6*, *PRL*, *SP1*, *STAT3*, *TLR4*, *TNF*	*CCK*, *NTN1*	*PRL*	*SMPD1*, *STAT3*	*CXCL12*, *SNCA*, *SP1*
**Molecules that downregulate *CTSB***		*TGFB1*	*VEGFA*			*IL10*
**Other relations**		*ANXA2*, *EGFR*, *HMGB1*, *MKI67*, *TP53*	*APOA1*, *KDR*, *PLAU*	*IL1B*	*CAV1*, *HGF*	*FOXO3*

**Table 7:** Most overrepresented GO biological processes that are common for the sets of genes linked with CTSB and associated with hyperglycemia, CVD, diabetic neuropathy, diabetic nephropathy, diabetic retinopathy, insulin resistance, and beta-cell dysfunction.

Gene Ontology Biological Process	Gene Ontology ID	*p*-Values with FDR Correction						
		Hyperglycemia	CVD	Diabetic Neuropathy	Diabetic Nephropathy	Diabetic Retinopathy	Insulin Resistance	Beta-Cell Dysfunction
Positive regulation of cell proliferation	GO:0008284	4.03 × 10^−9^	1.92 × 10^−5^	0.0011	2.85 × 10^−10^	6.20 × 10^−9^	1.92 × 10^−11^	1.03 × 10^−4^
Positive regulation of gene expression	GO:0010628	5.24 × 10^−10^	4.48 × 10^−8^	7.49 × 10^−8^	1.68 × 10^−11^	5.09 × 10^−10^	4.75 × 10^−11^	0.006
Positive regulation of protein phosphorylation	GO:0001934	8.99 × 10^−9^	8.17 × 10^−7^	2.68*10^−4^	1.31 × 10^−8^	6.19 × 10^−8^	6.81 × 10^−8^	0.0062
Lipopolysaccharide-mediated signaling pathway	GO:0031663	2.08 × 10^−6^	4.81 × 10^−6^	6.83 × 10^−4^	2.57 × 10^−5^	5.29 × 10^−6^	3.06 × 10^−7^	0.0062
Negative regulation of apoptotic process	GO:0043066	3.53 × 10^−9^	1.75 × 10^−6^	0.0011	3.36 × 10^−13^	2.88 × 10^−7^	4.87 × 10^−11^	0.006
Positive regulation of glial cell proliferation	GO:0060252	5.50 × 10^−6^	2.71 × 10^−7^	0.0052	3.82 × 10^−5^	1.23 × 10^−5^	1.13 × 10^−7^	0.0031
Protein kinase B signaling	GO:0043491	3.02 × 10^−6^	2.13 × 10^−4^	8.72 × 10^−4^	1.63 × 10^−6^	8.27 × 10^−6^	1.93 × 10^−4^	0.0074
Response to drug	GO:0042493	1.38 × 10^−9^	1.20 × 10^−6^	0.0072	3.64 × 10^−6^	9.35 × 10^−5^	6.81 × 10^−8^	0.0017
Positive regulation of protein kinase B signaling	GO:0051897	2.83 × 10^−5^	4.95 × 10^−7^	0.0085	2.60 × 10^−8^	1.51 × 10^−9^	6.81 × 10^−8^	0.0031
Positive regulation of interleukin-8 production	GO:0032757	2.09 × 10^−5^	6.03 × 10^−9^	0.002	9.11 × 10^−7^	3.52 × 10^−9^	7.49 × 10^−11^	0.0139

**Table 8:** Types of associations of the genes/proteins from hyperglycemia-related and cathepsin L (CTSL)-related networks with high glucose (HG) and cathepsin L (*STSL*).

	HG	Gene Expression Is Upregulated by HG	Gene Expression Is Downregulated by HG	Molecules with Hyperglycemic Activity	Molecules with Antihyperglycemic Effect	Other Relations
CTSL						
**Genes are upregulated by cathepsin L**		*BCL2*, *CXCL8*, *HPSE*	*BCL2*			
**Genes are downregulated by cathepsin L**		*CDKN1A*, *LEPR*	*CDH1*	*IGFBP3*		*LEPR*, *TF*
**Molecules that upregulate *CTSL***		*FGF2*, *FOXO1*, *HPSE*, *IL6*, *JUN*, *MAPK1*			*INS*, *MYC*	*FOS*
**Molecules that downregulate *CTSL***		*CDKN1A*, *TGFB1*				
**Other relations**		*CCL2*, *F3*, *TP53*	*PLAU*			*POMC*

**Table 9:** Most overrepresented GO biological processes that are common for the sets of genes linked with CTSL and associated with hyperglycemia, CVD, diabetic neuropathy, diabetic nephropathy, diabetic retinopathy, and insulin resistance.

Gene Ontology Biological Process	Gene Ontology ID	*p*-Values with FDR Correction					
		Hyperglycemia	CVD	Diabetic Neuropathy	Diabetic Nephropathy	Diabetic Retinopathy	Insulin Resistance
Positive regulation of gene expression	GO:0010628	7.19 × 10^−6^	0.0012	0.003	7.98 × 10^−4^	1.25 × 10^−4^	4.44 × 10^−4^
Chemotaxis	GO:0006935	8.06 × 10^−4^	0.0033		0.0021	0.0027	0.0146
Positive regulation of protein phosphorylation	GO:0001934			0.0167	0.0115	1.25 × 10^−4^	5.80 × 10^−4^
Positive regulation of MAPK cascade	GO:0043410	0.0165	0.0069	0.012	0.006	4.87 × 10^−5^	0.0086
Negative regulation of cell proliferation	GO:0008285	5.96 × 10^−5^	0.0414	0.0123	6.86 × 10^−4^		0.0041
Positive regulation of protein kinase B signaling	GO:0051897	8.49 × 10^−4^	0.023		0.0184	2.07 × 10^−4^	0.0163
Positive regulation of cell migration	GO:0030335	0.0058	0.0271	0.026		0.0027	
Lipopolysaccharide-mediated signaling pathway	GO:0031663	0.0173	0.0128		0.0103		0.033
Positive regulation of protein import into nucleus	GO:0042307	0.0195	0.0167		7.98 × 10^−4^		0.0438
Positive regulation of production of miRNAs involved in gene silencing by miRNA	GO:1903800	0.0034	0.0271	0.0039	0.0228		0.048

**Table 10:** Overrepresented GO biological processes for SARS-CoV-2-targeted human proteins found by DAVID (*p*-values < 0.05 with FDR correction).

Gene Ontology Biological Process	Gene Ontology ID	Genes	*p*-Values with FDR Correction
Viral process	GO:0016032	*BRD4*, *CCDC86*, *CRTC3*, *CUL2*, *EIF4H*, *ELOC*, *MFGE8*, *NLRX1*, *NUP210*, *NUP214*, *NUP54*, *NUP58*, *NUP62*, *NUP88*, *NUP98*, *POLA1*, *RAE1*, *RALA*, *RBX1*, *RHOA*	0.0011
Intracellular transport of virus	GO:0075733	*NUP210*, *NUP214*, *NUP54*, *NUP58*, *NUP62*, *NUP88*, *NUP98*, *RAE1*	0.0079
Protein transport	GO:0015031	*AKAP8*, *ARF6*, *CENPF*, *CHMP2A*, *ERC1*, *GORASP1*, *HOOK1*, *JAKMIP1*, *LMAN2*, *NUP210*, *PLEKHF2*, *PPT1*, *RAB14*, *RAB18*, *RAB2A*, *RAB5C*, *RAB7A*, *TIMM10B*, *TIMM8B*, *TIMM9*, *TMED5*, *WASHC4*, *YIF1A*	0.0013
Protein folding	GO:0006457	*BAG5*, *CSNK2A2*, *CSNK2B*, *CWC27*, *DNAJC19*, *ERO1B*, *ERP44*, *FKBP10*, *FKBP15*, *FKBP7*, *GNB1*, *GRPEL1*, *MOGS*, *PPIL3*, *QSOX2*, *SIL1*, *TBCA*	4.05 × 10^−4^
Regulation of glucose transport	GO:0010827	*NUP210*, *NUP214*, *NUP54*, *NUP58*, *NUP62*, *NUP88*, *NUP98*, *RAE1*	7.74 × 10^−4^
Mitotic nuclear envelope disassembly	GO:0007077	*NEK9*, *NUP210*, *NUP214*, *NUP54*, *NUP58*, *NUP62*, *NUP88*, *NUP98*, *RAE1*	7.74 × 10^−4^
tRNA export from nucleus	GO:0006409	*NUP210*, *NUP214*, *NUP54*, *NUP58*, *NUP62*, *NUP88*, *NUP98*, *RAE1*	7.74 × 10^−4^
Regulation of cellular response to heat	GO:1900034	*BAG5*, *HSBP1*, *NUP210*, *NUP214*, *NUP54*, *NUP58*, *NUP62*, *NUP88*, *NUP98*, *RAE1*	0.0022
G2/M transition of mitotic cell cycle	GO:0000086	*AKAP9*, *CDK5RAP2*, *CEP135*, *CEP250*, *CEP43*, *CIT*, *CNTRL*, *NINL*, *PCNT*, *PRKACA*, *PRKAR2B*, *RAB8A*	0.0087
Protein heterotrimerization	GO:0070208	*COL6A1*, *GNB1*, *NUP54*, *NUP58*, *NUP62*	0.0142
mRNA export from nucleus	GO:0006406	*NUP210*, *NUP214*, *NUP54*, *NUP58*, *NUP62*, *NUP88*, *NUP98*, *RAE1*, *SLU7*, *UPF1*	0.0142
U4 snRNA 3’-end processing	GO:0034475	*EXOSC2*, *EXOSC3*, *EXOSC5*, *EXOSC8*	0.0387
Chaperone-mediated protein transport	GO:0072321	*TIMM10*, *TIMM8B*, *TIMM9*, *TOR1A*	0.0387
Protein targeting to mitochondrion	GO:0006626	*DNAJC19*, *TIMM10*, *TIMM10B*, *TIMM8B*, *TIMM9*, *TOMM70*	0.0418
Nuclear-transcribed mRNA catabolic process, exonucleolytic, 3’-5’	GO:0034427	*EXOSC2*, *EXOSC3*, *EXOSC5*, *EXOSC8*	0.0497

**Table 11:** Most overrepresented GO biological processes for the sets of genes linked with SARS-CoV-2-targeted human proteins and associated with CVD, diabetic neuropathy, diabetic nephropathy, and diabetic retinopathy.

Gene Ontology Biological Process	Gene Ontology ID	*p*-Values with FDR Correction			
		CVD	Diabetic Neuropathy	Diabetic Nephropathy	Diabetic Retinopathy
Cytokine-mediated signaling pathway	GO:0019221	3.79 × 10^−23^	1.92 × 10^−11^	4.53 × 10^−37^	2.35 × 10^−20^
Response to hypoxia	GO:0001666	3.16 × 10^−16^	7.16 × 10^−10^	3.50 × 10^−27^	5.42 × 10^−25^
Positive regulation of gene expression	GO:0010628	1.25 × 10^−18^	1.71 × 10^−9^	1.59 × 10^−30^	2.56 × 10^−24^
Positive regulation of phosphatidylinositol 3-kinase signaling	GO:0014068	4.48 × 10^−17^	1.89 × 10^−8^	2.91 × 10^−24^	7.80 × 10^−22^
Inflammatory response	GO:0006954	7.23 × 10^−25^		1.53 × 10^−33^	3.86 × 10^−26^
Positive regulation of cell proliferation	GO:0008284		1.86 × 10^−8^	6.54 × 10^−25^	1.09 × 10^−22^
Positive regulation of smooth muscle cell proliferation	GO:0048661	1.01 × 10^−16^	2.09 × 10^−8^	8.79 × 10^−23^	
Positive regulation of protein kinase B signaling	GO:0051897	2.57 × 10^−16^		4.87 × 10^−23^	
Aging	GO:0007568	1.14 × 10^−16^	9.23 × 10^−11^		
Positive regulation of ERK1 and ERK2 cascade	GO:0070374		6.15 × 10^−9^	6.70 × 10^−24^	
Negative regulation of apoptotic process	GO:0043066			4.70 × 10^−28^	
Positive regulation of angiogenesis	GO:0045766				1.47 × 10^−25^
Cellular response to lipopolysaccharide	GO:0071222	3.00 × 10^−19^			
Positive regulation of protein phosphorylation	GO:0001934				4.30 × 10^−19^
Positive regulation of cell migration	GO:0030335				5.16 × 10^−19^
Response to xenobiotic stimulus	GO:0009410				3.10 × 10^−17^
Regulation of blood pressure	GO:0008217	3.16 × 10^−16^			
Positive regulation of peptidyl-tyrosine phosphorylation	GO:0050731		1.71 × 10^−9^		
Positive regulation of apoptotic process	GO:0043065		6.15 × 10^−9^		

## Data Availability

The data supporting the reported results are available in the Supplementary Materials.

## Supplementary Materials

The following supporting information can be downloaded at: https://www.mdpi.com/article/10.3390/ijms23137247/s1.

## Abbreviations

ACE	Angiotensin-converting enzyme
AR	Androgen receptor
CTS	Crosstalk specificity
CTSB	Cathepsin B
CTSL	Cathepsin L
CVD	Cardiovascular disease
DPP-4	Dipeptidyl peptidase-4
EGF	Epidermal growth factor
ERK	Extracellular signal-regulated kinase
GDF15	Growth differentiation factor 15
GO	Gene ontology
GPX1	Glutathione peroxidase 1
HG	High glucose
HMGB1	High-mobility group protein B1
HO-1	Heme oxygenase 1
IDF	Insulin-degrading enzyme
MAPK	Mitogen-activated protein kinase
PPARγ	Peroxisome proliferator-activated receptor gamma
RhoA	Ras homolog family member A
TGF-β	Transforming growth factor beta
TMPRSS2	Transmembrane protease, serine 2
TNF	Tumor necrosis factor
tPA	Tissue-type plasminogen activator
UCP2	Uncoupling protein 2
